# Pre-electrolysis of LiClO_4_ in Acetonitrile: Electrochemically Induced Protolytic Carbon–Carbon Bond Formation of Benzylic Ethers and Acetals with Allyl Trimethylsilane and Other Carbon Nucleophiles

**DOI:** 10.1021/acs.joc.3c01256

**Published:** 2023-08-18

**Authors:** Cornelius Fastie, Luomo Li, Moritz Bätcher, Gerhard Hilt

**Affiliations:** Institut für Chemie, Universität Oldenburg, Carl-von-Ossietzky-Str. 9-11, Oldenburg D-26129, Germany

## Abstract

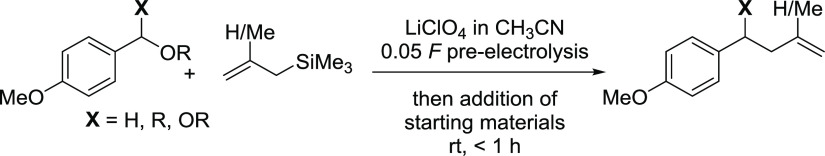

The pre-electrolysis of LiClO_4_ in acetonitrile in an undivided cell applying only “catalytic” amounts of current (e.g., 0.05 F) led to the formation of a strong acidic medium for the activation of benzylic ethers and acetals. The activated primary and secondary benzylic ethers and acetals could be converted with a range of carbon nucleophiles, such as allyl trimethylsilane, silyl enol ethers, and enol acetates, for the formation of new carbon–carbon bonds. A chemoselective reaction was observed when electron-deficient benzylic acetals were converted with allyl trimethylsilane to the monoallylated products, whereas an electron-rich benzylic acetal led to the double allylated product under activation of both ether groups.

## Introduction

**Scheme 1:**
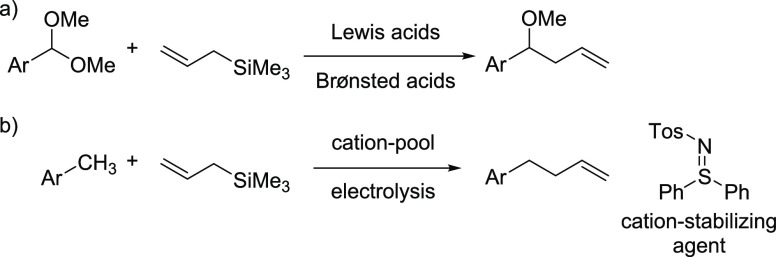
(a,b) Allylation Reactions in the Benzylic Position

Benzylic ethers and benzylic acetals are well-established protecting groups in organic synthesis. On the other hand, these functional groups can be utilized with neutral carbon nucleophiles, such as allyl trimethylsilanes and silyl enol ethers, for the formation of new carbon–carbon bonds. The stabilization of the primary carbenium ion-type intermediate is thereby accomplished with additional alkyl or aryl substituents in the benzylic position (= secondary or tertiary benzylic ethers), while purely primary benzylic ethers are quite robust and much harder to activate. However, a few reports describe the activation of such primary benzylic alcohols, ethers, and acetals utilizing Lewis acids such as FeCl_3_, In(OTf), and Sc(OTf)_3_.^1^ Additionally, the synthesis of homoallylic ethers can be accomplished by the Hosomi–Sakurai reaction when benzylic acetals and ketals are reacted with allyl trimethylsilane under Lewis acid catalysis. Among the Lewis acids established to undergo this transformation, a vast number of main group and several transition metal catalysts were reported. Among those are FeCl_3_,^2^ AlCl_3_,^3^ AlBr_3_/CuBr,^4^ BiBr_3_,^5^ Bi(OTf)_3_,^6^ Sc(OTf)_3_,^7^ NbCl_5_/AgClO_4_,^8^ Cp_2_Ti(SO_3_CF_3_)_2_,^9^ TiCl_4_,^10^ and Re(BrCO)_5_^11^ as well as silicon-, boron-, and carbon-based Lewis acids, such as TMSOTf,^12^ TMSNTf_2_,^13^ TMSN(SO_2_F)_2_,^9,14^ TMSI,^15^ BF_3_·Et_2_O,^3,16^ diphenylboryl triflate,^17^ and trityl perchlorate^17^ (Scheme 1a). Also, (chiral) organic Brønsted acids have been reported for the Hosomi–Sakurai reaction,^18,19^ and strong inorganic acids, such as HClO_4_ absorbed on silica,^20^ were also in the focus of interest. All of these reagents and catalysts have their advantages and disadvantages, but the Hosomi–Sakurai reaction is considered a redox-neutral reaction, and from an organic electrochemical point of view, there is obviously no report that electrochemical methods have been applied to facilitate this transformation. Accordingly, we would like to report herein the pre-electrolysis of the electrolyte for the simple application of an *in situ* generated strong Brønsted acid as a catalyst in the Hosomi–Sakurai reaction and similar transformations.

Also of interest in this respect are the electrochemical oxidation of toluene derivatives and the stabilization of the benzylic cation by a tosylated sulfanimine derivative in a cation-pool electrolysis as was described by Yoshida et al. (Scheme 1b).^21^

## Results and Discussion

**Scheme 2:**
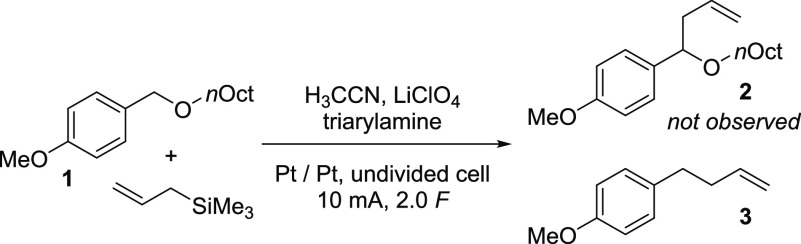
Attempted Electrochemical Allylation of Benzylic Ethers

The application of electrochemical methods is a hot topic in organic synthesis and related fields at current times, and a number of recent reviews are trying to keep up with the steadily increasing number of new transformations.^22^ In this respect, we focused our attention toward the activation of carbon–hydrogen bonds. In an attempt to oxidize benzylic ethers, such as **1**, under mild redox-mediated conditions, utilizing triarylamines as a catalyst, in the presence of the allyl trimethylsilane as an allylating agent, we attempted to access homoallylic ethers in a single transformation (Scheme 2).^23^ To our disappointment, the formation of the desired homoallylic ether **2** was not observed, but fortunately instead, the deoxygenated product **3** was formed in a control experiment in the absence of the triarylamine catalyst in substantial amounts.

The octyl ether in **1** was utilized to identify the side products derived from the “leaving group” 1-octanol by GC and GCMS analysis. As it turned out, 1-octanol could be detected alongside the octyl trimethylsilyl ether as side-products. This analysis of the reaction products revealed a redox-neutral transformation for the synthesis of **3** since no net redox process occurred. Therefore, we investigated this transformation in some more detail to determine the role of the electricity in this transformation. Over the course of the investigation, we identified conditions where only a catalytic amount of electricity (e.g., 0.025 F) was sufficient to realize the transformation. The hypothesis that an electrocatalytic process, where a radical cation is generated at the anode and the “hole” located at the starting material acts as catalyst in solution, was disproven when the pure electrolyte H_3_CCN/LiClO_4_ was electrolyzed applying a 0.05 *F* current and the starting materials were added after the electrolysis leading to complete conversion. After pre-electrolysis, the solution was colorless but turned to a fade-red color for a few seconds after the addition of the starting materials; after 5 min, the transformation was complete, and the product **3** was isolated in 86% yield. In the following, we tested a number of other solvent/supporting electrolyte combinations to realize the transformation. Only two other combinations, namely, H_3_CNO_2_/LiClO_4_ and H_3_CCN/NaClO_4_, gave the desired product **3** in significant amounts after pre-electrolysis towards utilizing a 0.05 *F* current in 75–80% yield, but the reaction time in nitromethane as a solvent was considerably longer (>4 h). Also, other supporting electrolytes, such as *n*Bu_4_NClO_4_ and KClO_4_ in acetonitrile, gave no or only trace amounts of product **3**. A selected number of key experiments are summarized in Table 1.

**Table 1:**
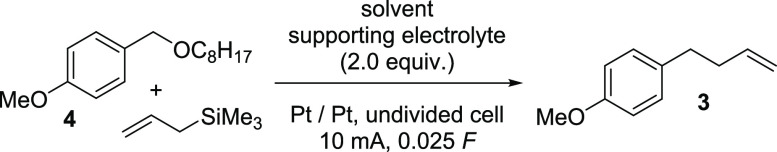
Results of the Pre-electrolysis of Solvent/Supporting Electrolyte Mixtures for the Synthesis of **3**^a^

no.	solvent	electrolyte^a^	yield of 3
1	CH_2_Cl_2_	LiClO_4_	0%
2	DMF	LiClO_4_	0%
3	2,2,2-trifluoroethanol	LiClO_4_	11%
4	CH_3_CN	LiClO_4_	86%^b^
5	CH_3_NO_2_	LiClO_4_	80%^c^
6	CH_3_CN	NaClO_4_	79%
7	CH_3_CN	KClO_4_	0%
8	CH_3_CN	Bu_4_NClO_4_	0%
9	CH_3_CN	Bu_4_NCl	0%

a The yields were determined by GC analysis with mesitylene as an internal standard, added after the reaction.

b Complete conversion after 5 min.

c Complete conversion after 4–5 h.

**Scheme 3:**
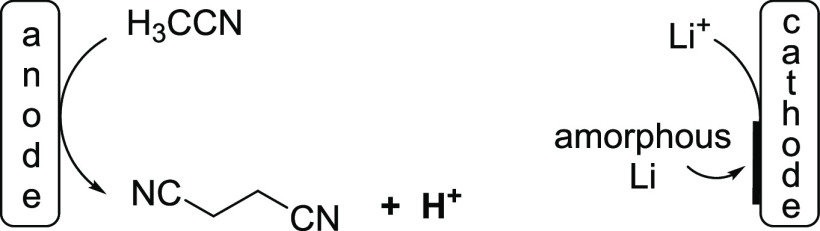
Proposed Reactions during the Pre-electrolysis of H_3_CCN/LiClO_4_

It seems that the combination of LiClO_4_ and acetonitrile generates a unique reactive medium for conducting this transformation, while other solvent/supporting electrolyte combinations mostly fail. This observation was already described by Torii et al. utilizing the combination of acetonitrile and LiClO_4_ under electrochemical conditions for other similar transformations.^24^ Interestingly, Torii and co-workers reported in their investigation of the ring opening of epoxides that chlorinated solvents, such as CH_2_Cl_2_ and ClCH_2_–CH_2_Cl, gave the best results, while the reaction in acetonitrile failed. However, when the pre-electrolysis of LiClO_4_ in acetonitrile was conducted in a divided cell applying 2.0 *F*, the anode compartment became very acidic, and in the GCMS spectrum, the formation of oxidation side products, such as succino nitrile (NC–CH_2_CH_2_–CN) as well as “higher oligomers”, was detected. At the cathode, the formation of a black precipitate was observed with the naked eyes. We propose that amorphous lithium was generated at the cathode, which reacted only very slowly with acetonitrile over time, and after the electrolysis, the electrodes were removed to obtain a highly acidic medium, and the lithium remained at the cathode (Scheme 3). By then, most of the organic transformations described herein were mostly complete. The black amorphous material on the cathode reacted with water under formation of gas, and therefore, we assume that the electrochemical pre-electrolysis primarily generated protons at the anode upon the oxidation of acetonitrile.

**Scheme 4:**
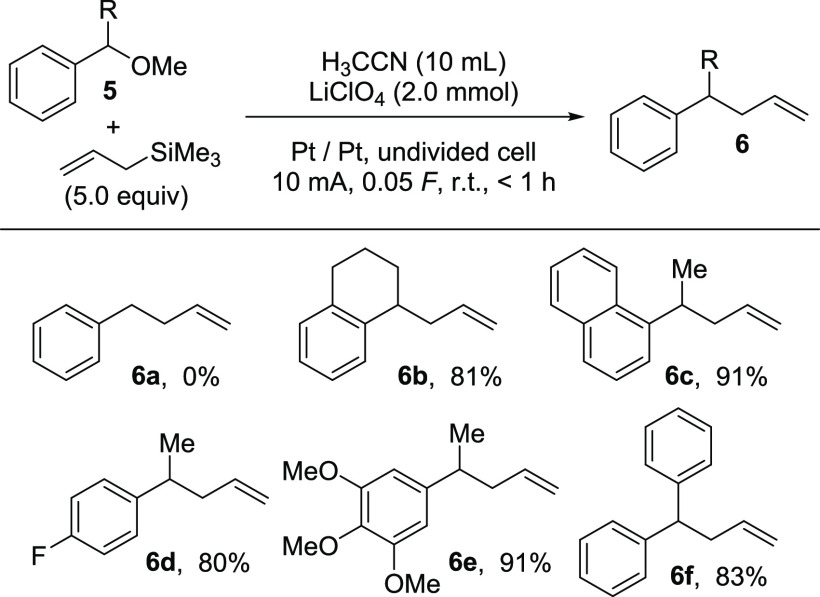
Application of the Pre-electrolysis of H_3_CCN/LiClO_4_ for the Allylation of Secondary Benzylic Ethers

When potassium or tetrabutyl ammonium perchlorate was tested as a supporting electrolyte, gas formation was observed at the cathode, no precipitate was formed at the cathode, and also no product formation could be detected. Accordingly, from HPLC-grade acetonitrile and easy-to-handle lithium perchlorate, a strong, water-free acid (= perchloric acid) was generated upon electrolysis, which induced the reactions that we report herein. Other reaction parameters, such as the applied amount of current, the electrode materials, the amount of the supporting electrolyte, the reaction temperature, the electrode distance, and the stirring rate, were also optimized (see the SI). With the optimized reaction conditions in hand, we investigated the scope and limitations of the benzylic ether activation of electrochemically *in situ* generated perchloric acid. For atom economic reasons, we also altered the “leaving group” from 1-octanol to methanol or ethanol in the further course of the investigation. The results of these reactions are summarized in Scheme 4.

For the activation of primary benzylic ether by the electrochemically generated HClO_4_, the 4-methoxy group in **4** seems to be necessary, as the desired product **6a** was not obtained. However, when secondary benzylic methoxy ethers were applied, desired products **6b**–**6f** were generated in good yields. The stabilization of the primary cationic intermediate, as proposed for similar Lewis acid-initiated processes, could be realized by alkyl (**6b**–**6e**) and an additional aryl group, as in **6f**. Noteworthy seems the fact that product **6d** was also generated in good yields although an electron-withdrawing fluoro substituent is in the 4-position.

**Scheme 5:**
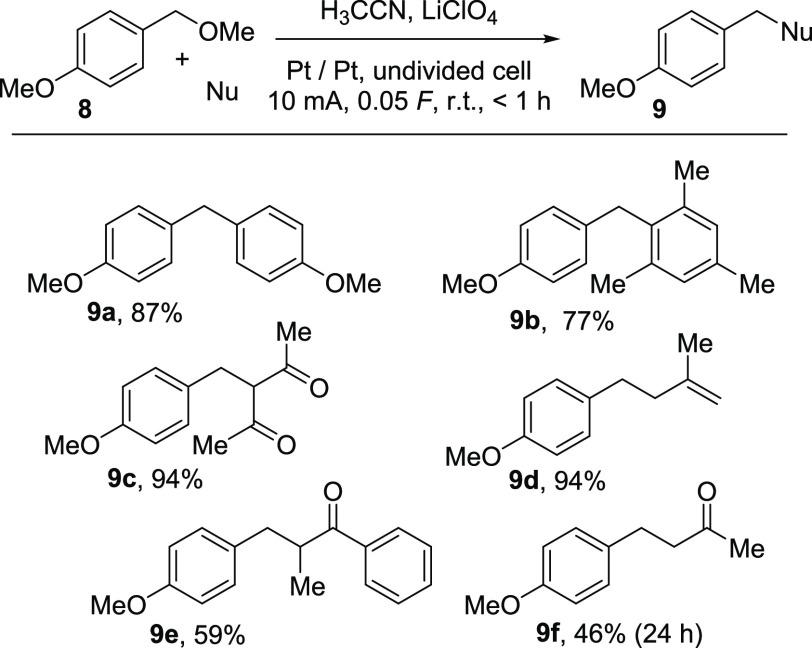
Application of the Pre-electrolysis of H_3_CCN/LiClO_4_ for the Nucleophilic Substitution of Benzylic Ethers

In order to test the compatibility of the transformation, we also tested other potential nucleophiles in the substitution reaction for the formation of carbon–carbon bonds. The results of these transformations are summarized in Scheme 5.

In this case, we used the benzylic ether **8** as an educt and added the starting materials after pre-electrolysis after consumption of 0.05 F. Electron-rich arenes gave the desired products **9a** and **9b** in terms of a Friedel–Crafts-type substitution in good yields,^25^ and product **9c** was formed in a very good yield of 94% from 2,4-pentadione as a mixture of tautomers. Allylation was also realized with trimethyl(2-methylallyl)silane to afford **9d** in a very good yield as well. Also, the substitution of an allyl silane with a silyl enol ether as a carbon nucleophile gave the desired carbon–carbon bond formation product **9e** in acceptable yields. In addition, the introduction of a nucleophilic acetone synthon for the synthesis of **9f** could be realized under the present reaction conditions when the silyl enol ether was substituted with prop-1-en-2-yl acetate to afford the desired product in 46% yield within 24 h, thereby expanding the scope of suitable carbon nucleophiles.

**Scheme 6:**
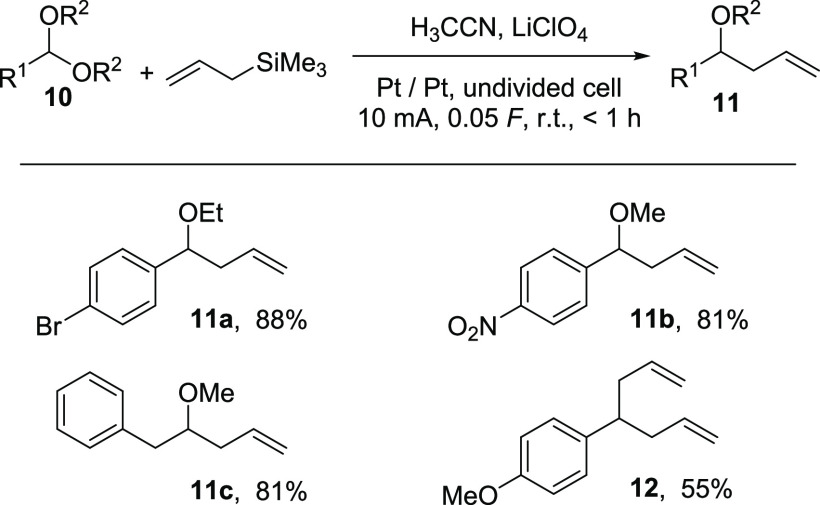
Application of the Pre-electrolysis of H_3_CCN/LiClO_4_ for the Allylation of Acetals

In the next set of experiments, the benzylic ethers were substituted by ethoxy and methoxy acetals of type **10** as this functional group can also be well-activated by Brønsted or Lewis acids and were reacted with allyl trimethyl silane. The results of these Hosomi–Sakurai transformations are summarized in Scheme 6.

The desired products of type **11** were generated in good yields of around 80% within a 1 h reaction time at ambient temperature. As in the case of secondary benzylic ethers, the aryl substituent could be substituted with strong electron-abstracting groups (**11b**), such as the 4-nitro substituent. Finally, a “homo”-benzylic acetal was reacted successfully to afford the product **11c** in 81% yield, which indicates that the substrate scope for this electrochemically generated reagent might be wider than we imagined. Also, the last reaction, applying the 4-methoxy-substituted benzylic acetal, led to a result that can be explained by the stabilization of the intermediate cation. The double benzylic methoxy substitution can be realized when an electron-rich arene is additionally stabilizing the cation derived from the corresponding monoallyl-substituted methoxy intermediate to afford product **12** in an acceptable yield of 55%.

In conclusion, we have explored a simple and easy-to-perform electrochemical method for the activation of primary electron-rich and secondary benzylic ethers as well as for benzylic acetals for carbon–carbon bond formation processes with allyl silanes and a range of other neutral carbon nucleophiles. The activation was made possible by pre-electrolysis of easy-to-handle lithium perchlorate in HPLC-grade acetonitrile in an undivided cell utilizing catalytic amounts of current (0.05 *F*) with respect to the ether/acetal starting materials.

## Experimental Section

### General Experimental Procedure for the Nucleophilic Substitution of Benzylic Ethers and Acetals

In an undivided cell, lithium perchlorate (213 mg, 2.0 mmol) was dissolved in acetonitrile (10 mL). Afterward, the mixture was electrolyzed under constant current (10 mA, 0.05 *F*) utilizing Pt plate electrodes (1.5 cm^2^). After removal of the electrodes, both the benzylic ether or acetal (1.0 mmol, 1.0 equiv) and the nucleophile (5.0 mmol, 5.0 equiv.; for acetals 10.0 mmol, 10.0 equiv) were added. Samples for GC-MS analysis were taken after 5 and 60 min. After completion of the reaction, an aqueous saturated Na_2_CO_3_ solution (5 mL) was added, and the mixture was extracted with Et_2_O (3 × 30 mL). The combined organic layers were dried over MgSO_4_ and filtered, and the solvent was removed under reduced pressure. The residue was purified by column chromatography to furnish the respective product.

Most of the generated products are literature known compounds, and the details for their specific synthesis, purification, and analytical data can be found in the Supporting Information.

## Data Availability

The data underlying this study are available in the published article and its Supporting Information

## Supporting Information Available

Synthesis, analytical data, and NMR spectra (PDF)
