# Supplementary material for: Pre-electrolysis of LiClO4 in Acetonitrile: Electrochemically Induced Protolytic Carbon–Carbon Bond Formation of Benzylic Ethers and Acetals with Allyl Trimethylsilane and Other Carbon Nucleophiles
Source: J Org Chem. 2023 Aug 18;88(17):12526–30. doi: 10.1021/acs.joc.3c01256 (PMC10476155; doi:10.1021/acs.joc.3c01256)
Supplement: Supplementary file 1 — jo3c01256_si_001.pdf [file jo3c01256_si_001.pdf]

Pre-Electrolysis of $LiClO_4$ in Acetonitrile: Electrochemically Induced Protolytic Carbon-Carbon Bond Formation of Benzylic Ethers and Acetals with Allyl Trimethylsilane and other Carbon Nucleophiles

Cornelius Fastie, Luomo Li, Moritz Bätcher and Gerhard Hilt*

Institut für Chemie, Universität Oldenburg, Carl-von-Ossietzky-Str. 9-11, D-26129 Oldenburg, Germany

1 General Information 1
2 Synthesis of the starting materials 3
2.1 Synthesis of benzyl ethers 3
2.2 Synthesis of acetals 3
2.3 Synthesis of 2-methylallyltrimethylsilane 4
3 Electrosynthesis 5
3.1 Optimization of the reaction conditions 5
3.2 General Procedure (GPC) for the nucleophilic substitution of benzylic ethers and acetals 6
3.3 Synthesized Products 7
4 NMR spectra of all synthesized compounds 17
5 References 33

1 General Information

All chemicals or reagents purchased from commercial suppliers were used without further purification, if not otherwise stated, or were prepared according to known literature procedures. If water or air sensitive compounds have been used, the experiments were carried out in heat gun dried glassware using conventional SCHLENK techniques under nitrogen atmosphere. Electrochemical reactions were carried out using an AIM-TTI Instruments MX100T *Triple Output Multi-Range DC Power Supply* (35 V, 3 A) or a *HMP4040 Programmable Power Supply 384 W* ROHDE&SCHWARZ (32 V, 10 A) power supply. These reactions were performed in an undivided cell equipped with a stirring bar, and platinum electrodes. All known compounds were characterized by ^{1}H and ^{13}C NMR and ^{19}F NMR if applicable. Unknown compounds were identified by ^{1}H NMR, ^{13}C NMR, ^{19}F NMR if applicable, IR and HRMS.

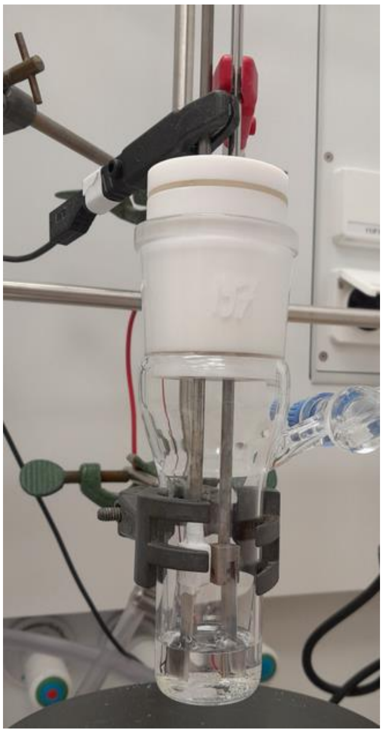

Figure S1: Undivided electrolysis cell, equipped with platinum electrodes.

NMR spectroscopy: NMR spectra were recorded either on a Bruker Avance 300 (300 MHz), on a Bruker Avance III (500 MHz) or on a Bruker Avance DRX (500 MHz). Chemical shifts are reported in parts per million (ppm). The spectra are referenced to the residual solvent peak of $CDCl_3$. In the ^{1}H NMR spectra this corresponds with the singlet of the solvent signal of $CDCl_3$ at δ = 7.26 ppm. The ^{13}C NMR spectra were referenced to the central line of the triplet of $CDCl_3$ at δ = 77.16 ppm. The stated form of the signal describes the appearance of the signal and not the theoretically expected form.

Infrared Spectroscopy: The IR spectra were obtained with a Shimadzu IRSpirit with a QATR-S cell. The wave numbers λ^{-1} are given in reciprocal centimeters (cm^{-1}).

Chromatography: Flash chromatography was carried out using MACHERY-NAGEL silica gel 60 (0.040-0.063 mm). Thin layer chromatography was carried out on MERCK TLC plates coated with silica gel 60 F_{254} with fluorescence indicator. For the detection of the signals ultraviolet light (λ = 254 nm) was used or heating after the plate has been dipped into a potassium permanganate-solution.

MS/HRMS: MS and HRMS spectra of products were obtained with a WATERS Q-TOF Premier (ESI, pos. mode or APCI) or THERMO SCIENTIFIC DFS (EI) spectrometers.

2 Synthesis of the starting materials

2.1 Synthesis of benzyl ethers

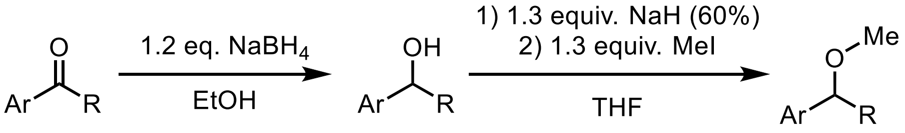

Most benzyl ethers were synthesized by combining an adapted literature procedure by FLEISCHER[1] for reducing carbonyl groups with another procedure by FAN[2] for ether synthesis. The ketone (10 mmol, 1.0 equiv.) was dissolved in ethanol (20 mL). The mixture was stirred vigorously while sodium borohydride (454 mg, 12.0 mmol, 1.2 equiv.) was slowly added. The conversion of the ketone was determined by GC-MS analysis. Afterwards, a solution of NH_4Cl (20 mL) was added carefully and the mixture was extracted with Et_2O (3 x 30 mL). The combined organic phases were dried over $MgSO_4$, filtered and concentrated *in vacuo*. Sufficient purity of the alcohol was confirmed by ^{1}H NMR spectroscopy. The benzyl alcohol was then dissolved in THF (20 mL) and cooled to 0 °C, before sodium hydride (60% in mineral oil, 520 mg, 13.0 mmol, 1.3 equiv.) was added. After stirring for 20 minutes at this temperature, methyl iodide (0.8 mL, 13.0 mmol, 1.3 equiv.) was added. The conversion of the alcohol was confirmed by GC-MS analysis. Afterwards, water (20 mL) was added slowly. The aqueous layer was extracted with Et_2O (3 x 30 mL) and the combined organic layers were dried over $MgSO_4$, filtered and concentrated *in vacuo*. The residue was purified by column chromatography to furnish the respective benzyl ether.

2.2 Synthesis of acetals

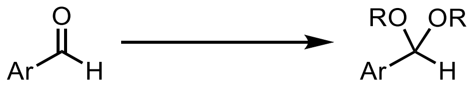

To a solution of the respective aldehyde (5.0 mmol) in the alcohol (ROH, 66 mL) trifluoroacetic acid (1 mol%) was added. This mixture was stirred for 30 minutes at room temperature and then a saturated aqueous $NaHCO_3$ solution (6 mL) was added. The aqueous layer was extracted with Et_2O (3 x 30 mL) and the combined organic layers were dried over $MgSO_4$. After filtration and removal of the solvent *in vacuo*, the residue was purified by column chromatography to furnish the respective acetals.

2.3 Synthesis of 2-methylallyltrimethylsilane

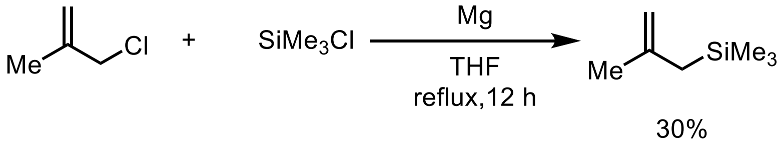

According to a procedure by MARKOVIC[3], magnesium turnings (7.3 g, 300 mmol, 2.0 equiv.) were suspended in anhydrous THF (20 mL) under an inert gas atmosphere. After adding a bead of iodine, a solution of trimethylsilyl chloride (16.3 g, 150 mmol, 1.0 equiv.) and (2-methyl)-allyl chloride (18.1 g, 200 mmol, 1.3 equiv.) in anhydrous THF (130 mL) was dropped into the suspension in a way, that the suspension was kept at reflux. Afterwards, the mixture was heated for another 12 h. After filtration, the excess solvent was removed *in vacuo*. The residue was distilled (220 mbar, 55 °C) and the product (5.7 g, 45 mmol, 30%) was obtained as a colourless oil.

^{1}H NMR (500 MHz, $CDCl_3$): δ = 4.58-4.57 (m, 1H), 4.47-4.45 (m, 1H), 1.71 (s, 3H), 1.53 (s, 2H), 0.02 (s, 9H) ppm.

^{13}C{1H} NMR (125 MHz, $CDCl_3$): δ = 144.1, 108.1, 28.8, 25.3, 1.2 ppm.

IR (ATR, neat): λ^{-1} = 3076 (w), 2956 (w), 2359 (w), 1739 (w), 1637 (w), 1374 (w), 1280 (w), 1249 (s), 1163 (w), 999 (w), 973 (w), 849 (s), 839 (s), 769 (s) 694 (m), 657 (w), 594 (w), 491 (m) cm^{-1}.

HRMS (EI): calcd. for $C_7H_{16}Si$ [M^+]: m/z = 128.1016; found m/z = 128.1018.

3 Electrosynthesis

3.1 Optimization of the reaction conditions

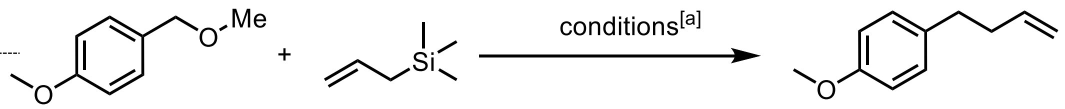

Entry	Change from conditions	Yield [%][b]
1	none	86
	Amount of current	
2	0.01 *F*	61
3	0.025 *F*	85
4	0.1 *F*	77
	Equivalents of allyltrimethylsilane	
5	1.0 equiv.	45
6	2.0 equiv.	55
7	3.0 equiv.	58
8	4.0 equiv.	66
9	7.0 equiv.	71
	Electrode material	
10	glassy carbon	79
11	stainless steel	0
12	graphite	0
	solvent	
13	DCM	0
14	acetone	0
15	DMF	0
16	2,2,2-trifluoroethanol	11
17	nitromethane	80[c]
	Supporting electrolyte	
18	$NaClO_4$	79
19	$KClO_4$	0
20	$n\mathrm{Bu_4NClO_4}$	0
21	$n\mathrm{Bu_4NCl}$	0

	Equivalents of supporting electrolyte	
22	0 equiv.	0
23	3.0 equiv.	74
24	4.0 equiv.	73
25	5.0 equiv.	77
	Temperature	
26	0 °C	15
27	50 °C	70
	Stirring velocity	
28	100 rpm	53
29	1000 rpm	68

[a] The reactions were performed in an undivided cell on a 0.50 mmol scale with a substrate concentration of 0.05 M. Conditions: 0.05 *F*, 5.0 equiv. allyltrimethylsilane, Pt electrodes, MeCN, 25 °C (ambient temperature), 10 mA, 2.0 equiv. $LiClO_4$, 335 rpm. [b] Yield determined by GC analysis of the unpurified reaction mixture with mesitylene as internal standard. [c] Reaction time 4-5 h.

3.2 General Procedure (GPC) for the nucleophilic substitution of benzylic ethers and acetals

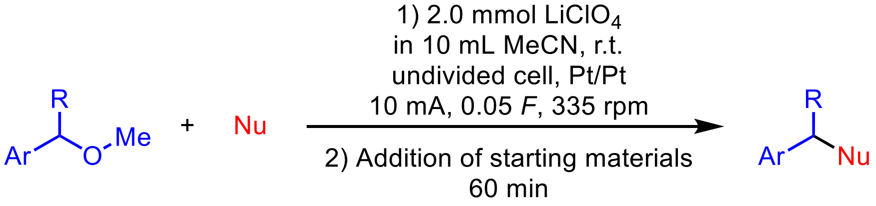

In an undivided cell lithium perchlorate (213 mg, 2.0 mmol) was dissolved in acetonitrile (10 mL). Afterwards, the mixture was electrolyzed under constant current (10 mA, 0.05 *F*) utilizing Pt plate electrodes (1.5 cm^2). After removal of the electrodes, both the benzylic ether or acetal (1.0 mmol, 1 equiv.) and the nucleophile (5.0 mmol, 5.0 equiv.; for acetals 10.0 mmol, 10.0 equiv.) were added. Samples for GC-MS analysis were taken after five and 60 minutes. After completion of the reaction, an aqueous saturated Na_2CO_3 solution (5 mL) was added and the mixture was extracted with Et_2O (3 x 30 mL). The combined organic layers were dried over $MgSO_4$, filtered and the solvent was removed under reduced pressure. The residue was purified by column chromatography to furnish the respective product.

3.3 Synthesized Products

4-(4-Methoxyphenyl)-but-1-ene (3)

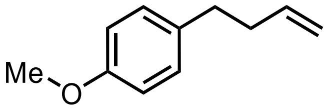

3

According to GPC, 4-methoxybenzylmethylether (152 mg, 1.0 mmol, 1.0 equiv.) and allyltrimethylsilane (571 mg, 5.0 mmol, 5.0 equiv.) were converted to furnish product **3** (136 mg, 0.84 mmol, 71%) as colourless oil after column chromatography (SiO_2, *dry load* on SiO_2, *n*-pentane:Et_2O = 20:1, R_f = 0.85).

^{1}H NMR (500 MHz, $CDCl_3$): δ = 7.13-7.09 (m, 2H), 6.85-6.82 (m, 2H), 5.90-5.82 (m, 1H), 5.06-5.02 (m, 1H), 4.99-4.96 (m, 1H), 3.78 (s, 3H), 2.68-2.64 (m, 2H), 2.37-2.33 (m, 2H) ppm.

^{13}C{1H} NMR (125 MHz, $CDCl_3$): δ = 158.0, 138.4, 134.2, 129.5, 115.0, 113.9, 55.4, 35.9, 34.7 ppm.

The spectroscopic data are in accordance with literature values.[4]

1-Allyl-1,2,3,4-tetrahydronaphthalene (6b)

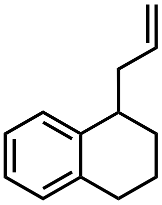

6b

According to GPC, 1-methoxy-1,2,3,4-tetrahydronaphthalene (162 mg, 1.0 mmol, 1.0 equiv.) and allyltrimethylsilane (571 mg, 5.0 mmol, 5.0 equiv.) were converted to furnish product **6b** (139 mg, 0.81 mmol, 81%) as colourless oil after column chromatography (SiO_2, *dry load* on SiO_2, *n*-pentane, R_f = 0.80).

^{1}**H NMR** (500 MHz, $CDCl_3$): δ = 7.24-7.22 (m, 1H), 7.16-7.10 (m, 3H), 5.92-5.84 (m, 1H) 5.11-5.06 (m, 2H), 2.91-2.89 (m, 1H), 2.80-2.78 (m, 2H), 2.56-2.51 (m, 1H,), 2.40-2.33 (m, 1H), 1.92-1.84 (m, 2H), 1.78-1.71 (m, 2H) ppm.

^{13}**C{1H} NMR** (125 MHz, $CDCl_3$): δ = 140.7, 137.6, 137.4, 129.2, 128.7, 125.7, 116.2, 41.3, 37.5, 29.9, 27.4, 19.9 ppm.

The spectroscopic data are in accordance with literature values.[5]

4-Naphthylpent-1-ene (6c)

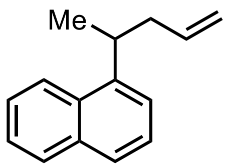

6c

According to GPC, 1-(1-methoxyethyl)-naphthalene (186 mg, 1.0 mmol, 1.0 equiv.) and allyltrimethylsilane (571 mg, 5.0 mmol, 5.0 equiv.) were converted to furnish product **6c** (178 mg, 0.91 mmol, 91%) as colourless oil after column chromatography (SiO_2, *dry load* on SiO_2, *n*-pentane, R_f = 0.38).

^{1}**H NMR** (500 MHz, $CDCl_3$): δ = 8.15 (d, ^3J = 8.4 Hz, 1H), 7.89 (d, ^3J = 8.0 Hz, 1H), 7.74 (d, ^3J = 8.0 Hz, 1H), 7.56-7.41 (m, 4H), 5.85 (ddt, ^3J = 17.0, 10.2, 6.9 Hz, 1H), 5.12-5.08 (m, 1H), 5.04-5.01 (m, 1H), 3,72 (sext, ^3J = 6.9 Hz, 1H), 2.67-2.61 (m, 1H), 2.48-2.41 (m, 1H), 1.43 (d, ^3J = 6.9 Hz, 1H) ppm.

^{13}**C{1H} NMR** (125 MHz, $CDCl_3$): δ = 143.1, 137.3, 134.1, 131.7, 129.1, 126.6, 125.9, 125.7, 125.4, 123.3, 122.7, 116.2, 42.1, 33.8, 21.1 ppm.

The spectroscopic data are in accordance with literature values.[5]

4-(4-Fluorobenzene)-pent-1-ene (6d)

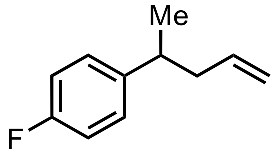

6d

According to GPC, 1-fluoro-4-(1-methoxyethyl)-benzene (154 mg, 1.0 mmol, 1.0 equiv.) and allyltrimethylsilane (571 mg, 5.0 mmol, 5.0 equiv.) were converted to furnish product **6d** (131 mg, 0.80 mmol, 80%) as colourless oil after column chromatography (SiO_2, *dry load* on SiO_2, *n*-pentane, R_f = 0.75).

^{1}H NMR (500 MHz, $CDCl_3$): δ = 7.17-7.14 (m, 2H), 7.00-6.97 (m, 2H), 5.70 (ddt, ^3J = 17.1, 10.2, 7.0 Hz, 1H), 5.02-4.96 (m, 2H), 2.80 (sext, ^3J = 7.0 Hz, 1H), 2.39-2.26 (m, 2H), 1.25 (d, ^3J = 6.9 Hz, 3H) ppm.

^{13}C{1H} NMR (125 MHz, $CDCl_3$): δ = 161.4 (d, ^1J = 243.2 Hz), 142.8 (d, ^4J = 3.2 Hz), 137.0, 128.4 (d, ^3J = 7.7 Hz), 116.2, 115.1 (d, ^2J = 20.9 Hz), 42.9, 39.3, 21.8 ppm.

^{19}F NMR (470 MHz, $CDCl_3$): δ = -117.7 ppm.

The spectroscopic data are in accordance with literature values.[6]

4-(3,4,5-Trimethoxybenzene)-pent-1-ene (6e)

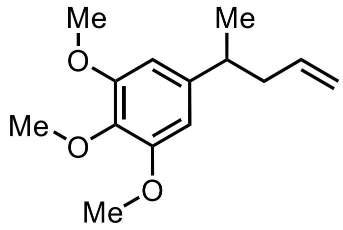

6e

According to GPC, 1,2,3-trimethoxy-5-(1-methoxyethyl)-benzene (226 mg, 1.0 mmol, 1.0 equiv.) and allyltrimethylsilane (571 mg, 5.0 mmol, 5.0 equiv.) were converted to furnish product **6e** (215 mg, 0.91 mmol, 91%) as a yellow oil after column chromatography (SiO_2, *dry load* on SiO_2, *n*-pentane:Et_2O = 2:1, R_f = 0.90).

^{1}**H NMR** (500 MHz, $CDCl_3$): δ = 6.41 (s, 2H), 5.77-5.68 (m, 1H), 5.04-4.97 (m, 2H), 3.85 (s, 6H), 3.83 (s, 3H), 2.72 (sext, ^3J = 7.1 Hz, 1H), 2.40-2.34 (m, 1H), 2-28-2.23 (m, 1H), 1.24 (d, ^3J = 7.0 Hz, 3H) ppm.

^{13}**C{1H} NMR** (125 MHz, $CDCl_3$): δ = 153.2, 143.0, 137.2, 136.4, 116.1, 104.2, 61.0, 56.3, 42.9, 40.3, 21.7 ppm.

IR: (ATR, neat): λ^{-1} = 2957 (m), 2927 (m), 2837 (w), 1739 (w), 1587 (s), 1510 (m), 1456 (m), 1419 (m), 1346 (m), 1321 (m), 1236 (s), 1184 (w), 1124 (s), 1010 (m), 911 (m), 826 (m), 776 (m), 663 (w), 531 (w) cm^{-1}.

HRMS (EI): calcd. for $C_{14}H_{20}O_3$ [M^+]: m/z = 236.1407; found m/z = 236.1404.

4,4-Diphenylbut-1-ene (6f)

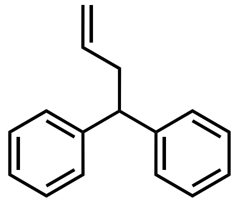

6f

According to GPC, benzhydrylmethylether (198 mg, 1.0 mmol, 1.0 equiv.) and allyltrimethylsilane (571 mg, 5.0 mmol, 5.0 equiv.) were converted to furnish product **6f** (173 mg, 0.83 mmol, 83%) as colourless oil after column chromatography (SiO_2, *dry load* on SiO_2, *n*-pentane, R_f = 0.38).

^{1}**H NMR** (500 MHz, $CDCl_3$): δ = 7.31-7.24 (m, 8H), 7.20-7.17 (m, 2H), 5.75 (ddt, *J* = 17.0, 10.2, 6.8 Hz, 1H), 5.07-5.03 (m, 1H), 4.98-4.95 (m, 1H), 4.03 (t, *J* = 7.9 Hz, 1H), 2.84 (ddt, *J* = 8.0, 6.8 ,1.4 Hz, 2H) ppm.

^{13}**C{1H} NMR** (125 MHz, $CDCl_3$): δ = 144.7, 137.0, 128.5, 128.1, 126.3, 116.4, 51.4, 40.1 ppm.

The spectroscopic data are in accordance with literature values.[5]

Di-(4-methoxyphenyl)-methane (9a)

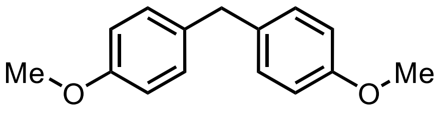

9a

According to GPC, 4-methoxybenzylmethylether (152 mg, 1.0 mmol, 1.0 equiv.) and anisole (541 mg, 5.0 mmol, 5.0 equiv.) were converted to furnish product **9a** (199 mg, 0.87 mmol, 87%) as colourless wax after dry column vacuum chromatography (SiO_2, *n*-heptane:ethyl acetate = 50:1, R_f = 0.20).

^{1}H NMR (500 MHz, $CDCl_3$): δ = 7.16-7.11 (m, 4H), 6.90-6.85 (m, 4H), 3.92-3.90 (m, 2H), 3.82-3.80 (m, 6H) ppm.

^{13}C{1H} NMR (125 MHz, $CDCl_3$): δ = 158.1, 133.8, 129.8, 114.0, 55.3, 40.3 ppm.

The spectroscopic data are in accordance with literature values.[4]

2-(4-Methoxybenzyl)-1,3,5-trimethylbenzene (9b)

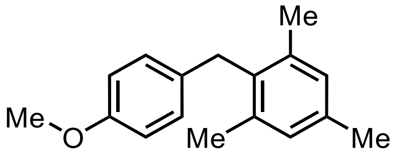

9b

According to GPC, 4-methoxybenzylmethylether (152 mg, 1.0 mmol, 1.0 equiv.) and mesitylene (601 mg, 5.0 mmol, 5.0 equiv.) were converted to furnish product **9b** (185 mg, 0.77 mmol, 77%) as colourless solid after column chromatography (SiO_2, *n*-pentane:Et_2O = 10:1, R_f = 0.60).

^{1}H NMR (300 MHz, $CDCl_3$): δ = 6.98-6.96 (m, 2H), 6.93 (s, 2H) 6.83-6.81 (m, 2H), 4.00 (s, 2H), 3.79 (s, 3H), 2.33 (s, 3H), 2.25 (s, 6H) ppm.

^{13}C{1H} NMR (125 MHz, $CDCl_3$): δ = 157.9, 137.1, 135.7, 134.3, 132.2, 129.0, 128.9, 113.9, 55.4, 33.9, 21.0, 20.2 ppm.

The spectroscopic data are in accordance with literature values.[7]

3-(4-Methoxybenzyl)-2,4-pentadione (9c)

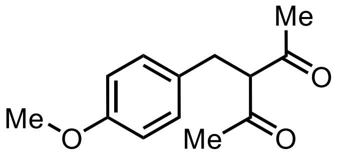

9c

According to GPC, 4-methoxybenzylmethylether (152 mg, 1.0 mmol, 1.0 equiv.) and 2,4-pentadione (506 mg, 5.0 mmol, 5.0 equiv.) were converted to furnish product **9c** (208 mg, 0.94 mmol, 94%) as a colourless solid after column chromatography (SiO_2, *dry load* on SiO_2, *n*-pentane:ethyl acetate = 50:1, R_f = 0.10). The product was obtained as a mixture of enol and keto tautomers. By integrating the ^{1}H NMR signals at 3.78 ppm and 3.76 ppm respectively, the ratio between enol and keto tautomer was determined to be 3:2.

^{1}H NMR (500 MHz, $CDCl_3$):

Enol tautomer: δ = 7.08-7.03 (m, 2H), 6.85-6.79 (m, 2H), 3.78 (s, 3H), 3.59 (s, 2H), 2.06 (s, 6H) ppm.

Keto tautomer: δ = 7.08-7.03 (m, 2H), 6.85-6.79 (m, 2H), 3.96 (t, J = 7.5 Hz, 1H) 3.76 (s, 3H), 3.09 (d, J = 7.5 Hz, 2H), 2.11 (s, 6H) ppm.

^{13}C{1H} NMR (125 MHz, $CDCl_3$): δ = 203.8, 191.9, 158.5, 158.3, 131.7, 130.1, 129.7, 128.4, 114.3, 114.2, 108.7, 70.3, 55.4, 55.3, 33.6, 32.2, 29.8, 23.3 ppm.

Mp.: 70-71 °C.

The spectroscopic data are in accordance with literature values.[4]

4-(4-Methoxyphenyl)-2-methyl-1-butene (9d)

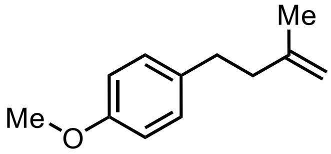

9d

According to GPC, 4-methoxybenzylmethylether (152 mg, 1.0 mmol, 1.0 equiv.) and 2-methylallyltrimethylsilane (641 mg, 5.0 mmol, 5.0 equiv.) were converted to furnish product

9d (166 mg, 0.94 mmol, 94%) as a colourless oil after column chromatography (SiO_2, *dry load* on SiO_2, *n*-pentane:Et_2O = 100:1, R_f = 0.30).

^{1}H NMR (500 MHz, $CDCl_3$): δ = 7.13-7.12 (m, 2H), 6.85-6.84 (m, 2H), 4.76-4.72 (m, 2H), 3.80 (s, 3H), 2.74-2.70 (m, 2H), 2.33-2.29 (m, 2H), 1.78 (s, 3H) ppm.

^{13}C{1H} NMR (125 MHz, $CDCl_3$): δ = 157.9, 146.6, 134.5, 129.3, 113.9, 110.3, 55.4, 40.0, 33.5, 22.7 ppm.

The spectroscopic data are in accordance with literature values.[5]

3-(Methoxyphenyl)-2-methyl-1-phenyl-1-propanone (9e)

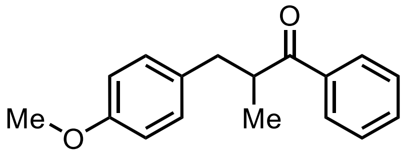

9e

According to GPC, 4-methoxybenzylmethylether (152 mg, 1.0 mmol, 1.0 equiv.) and trimethylsiloxy-1-phenyl-1-butene (1032 mg, 5.0 mmol, 5.0 equiv.) were converted to furnish product **9e** (151 mg, 0.59 mmol, 59%) as a yellowish oil after column chromatography (SiO_2, *dry load* on SiO_2, *n*-pentane:Et_2O = 10:1, R_f = 0.30).

^{1}H NMR (500 MHz, $CDCl_3$): δ = 7.93-7.91 (m, 2H), 7.56-7.52 (m, 1H), 7.46-7.43 (m, 2H), 7.13-7.10 (m, 2H), 6.82-6.79 (m, 2H), 3.77 (s, 3H), 3.77-3.69 (m, 1H), 3.11 (dd, ^3J = 6.4 Hz, ^2J = 13.8 Hz, 1H), 2.64 (dd, ^3J = 7.7 Hz, ^2J = 13.8 Hz, 1H), 1.20 (d, ^3J = 6.9 Hz, 3H) ppm.

^{13}C{1H} NMR (125 MHz, $CDCl_3$): δ = 204.0, 158.2, 136.8, 133.0, 132.2, 130.2, 128.8, 128.4, 114.0, 55.4, 43.1, 38.7, 17.5 ppm.

The spectroscopic data are in accordance with literature values.[8]

4-(4-Methoxyphenyl)-2-butanone (9f)

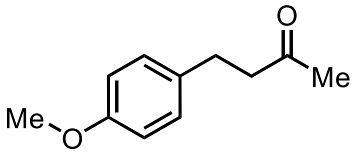

9f

According to GPC, 4-methoxybenzylmethylether (152 mg, 1.0 mmol, 1.0 equiv.) and ispropenyl acetate (501 mg, 5.0 mmol, 5.0 equiv.) were converted to furnish product **9e** (82 mg, 0.46 mmol, 46%) as a yellowish oil after column chromatography (SiO_2, *dry load* on SiO_2, *n*-pentane:Et_2O = 9:1, R_f = 0.15).

^{1}H NMR (500 MHz, $CDCl_3$): δ = 7.11-7.09 (m, 2H), 6.83-6.81 (m, 2H), 3.78 (s, 3H), 2.86-2.83 (m, 2H), 2.74-2.71 (m, 2H), 2.13 (s, 3H) ppm.

^{13}C{1H} NMR (125 MHz, $CDCl_3$): δ = 208.2, 158.2, 133.2, 129.4, 114.1, 55.4, 45.6, 30.2, 29.1 ppm.

The spectroscopic data are in accordance with literature values.[9]

1-Bromo-4-(1-ethoxybut-3-en)-benzene (11a)

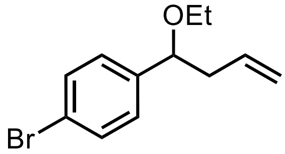

11a

According to GPC, 4-bromo-1-(diethoxymethyl)benzene (259 mg, 1.0 mmol, 1.0 equiv.) and allyltrimethylsilane (1143 mg, 10.0 mmol, 10.0 equiv.) were converted to furnish product **11a** (224 mg, 0.88 mmol, 88%) as a colourless oil after column chromatography (SiO_2, *dry load* on SiO_2, *n*-pentane:ethyl acetate = 100:1, R_f = 0.15).

^{1}H NMR (500 MHz, $CDCl_3$): δ = 7.47-7.45 (m, 2H), 7.18-7.17 (m, 2H), 5.74 (ddt, J = 17.2, 10.4. 7.0 Hz, 1H), 5.05-4.99 (m, 2H), 4.24-4.21 (m, 1H), 3.38-3.31 (m, 2H), 2.57-2.51 (m, 1H), 2.39-2.33 (m, 1H), 1.17 (t, J = 7.0 Hz, 3H) ppm.

^{13}C{1H} NMR (125 MHz, $CDCl_3$): δ = 141.7, 134.6, 131.6, 128.5, 121.3, 117.2, 81.3, 64.4, 42.6, 15.4 ppm.

The spectroscopic data are in accordance with literature values.[10]

1-(1-Methoxybut-3-ene)-4-nitrobenzene (11b)

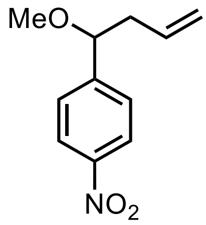

11b

According to GPC, 4-nitrobenzaldehydedimethyl acetal (197 mg, 1.0 mmol, 1.0 equiv.) and allyltrimethylsilane (1143 mg, 10.0 mmol, 10.0 equiv.) were converted to furnish product **11b** (168 mg, 0.81 mmol, 81%) as a colourless oil.

^{1}H NMR (500 MHz, $CDCl_3$): δ = 8.23-8.19 (m, 2H), 7.47-7.44 (m, 2H), 5.77-5.68 (m, 1H), 5.06-5.00 (m, 2H), 4.29 (t, J = 5.5 Hz, 1H), 3.26 (s, 3H), 2.58-2.38 (m, 2H) ppm.

^{13}C{1H} NMR (125 MHz, $CDCl_3$): δ = 149.6, 147.7, 133.6, 127.6, 123.8, 118.0, 82.9, 57.3, 42.3 ppm.

The spectroscopic data are in accordance with literature values.[11]

4-Methoxy-5-phenyl-1-pentene (11c)

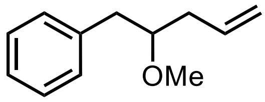

11c

According to GPC, 2-phenyl-1,3-dioxolane (166 mg, 1.0 mmol, 1.0 equiv.) and allyltrimethylsilane (1143 mg, 10.0 mmol, 10.0 equiv.) were converted to furnish product **11c** (144 mg, 0.81 mmol, 81%) as a yellowish oil.

^{1}**H NMR** (500 MHz, $CDCl_3$): δ = 7.34-7.31 (m, 2H), 7.30-7.23 (m, 3H), 5.95-5.86 (m, 1H), 5.15-5.13 (m, 1H), 5.12-5.10 (m, 1H), 3.50 (pent, J = 6.0 Hz, 1H), 3.37 (s, 3H), 2.88 (dd, J = 13.9, 6.5 Hz, 1H), 2.80 (dd, J = 13.9, 6.0 Hz, 1H), 2.35-2.24 (m, 2H) ppm.

^{13}**C{1H} NMR** (125 MHz, $CDCl_3$): δ = 139.1, 134.9, 129.6, 128.4, 126.2, 117.3, 82.0, 57.2, 40.1, 37.8 ppm.

The spectroscopic data are in accordance with literature values.[12]

4-(4-Methoxybenzene)-hepta-1,6-diene (12)

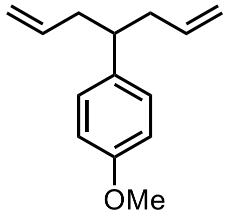

12

According to GPC, 4-methoxybenzaldehydedimethyl acetal (182 mg, 1.0 mmol, 1.0 equiv.) and allyltrimethylsilane (1143 mg, 10.0 mmol, 10.0 equiv.) were converted to furnish product **12** (144 mg, 0.81 mmol, 81%) as a colourless oil after column chromatography (SiO_2, *n*-pentane, R_f = 0.10).

^{1}**H NMR** (500 MHz, $CDCl_3$): δ = 7.09-7.06 (m, 2H), 6.85-6.82 (m, 2H), 5.71-5.63 (m, 2H), 4.99-4.92 (m, 4H), 3.79 (s, 3H), 2.67-2.66 (m, 1H), 2.43-2.28 (m, 4H) ppm.

^{13}**C{1H} NMR** (75 MHz, $CDCl_3$): δ = 158.1, 137.1, 136.9, 128.7, 116.1, 113.8, 55.4, 44.9, 40.6 ppm.

The spectroscopic data are in accordance with literature values.[13]

4 NMR spectra of all synthesized compounds

1-Allyl-1,2,3,4-tetrahydronaphthalene (6b)

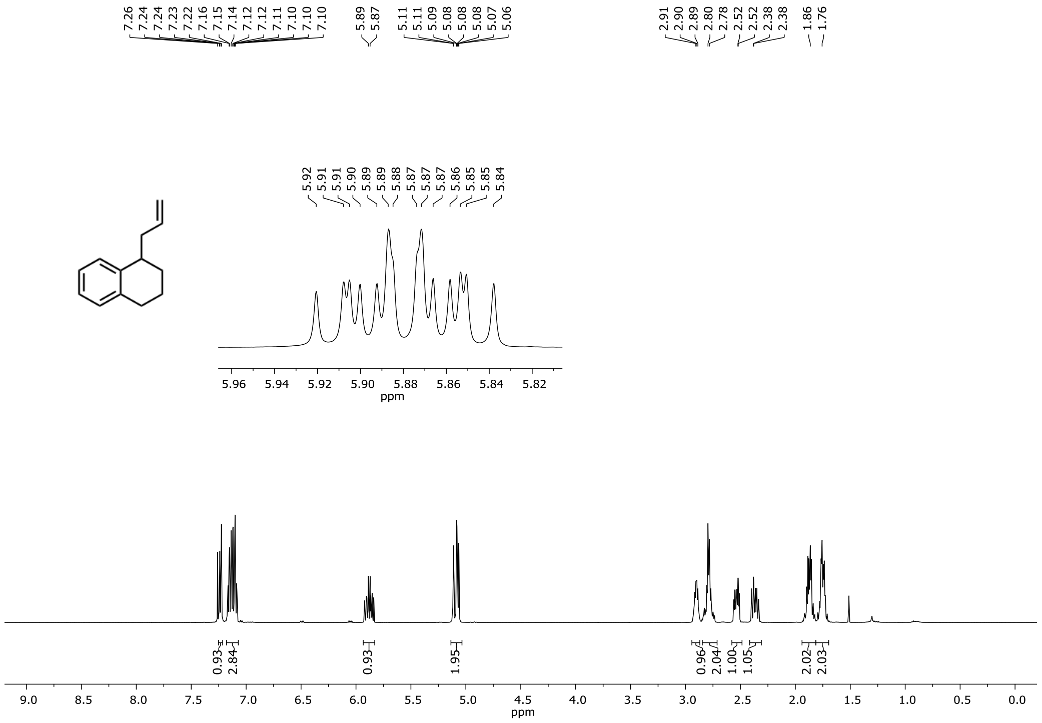

^{1}H NMR (500 MHz, $CDCl_3$) of compound **6b**.

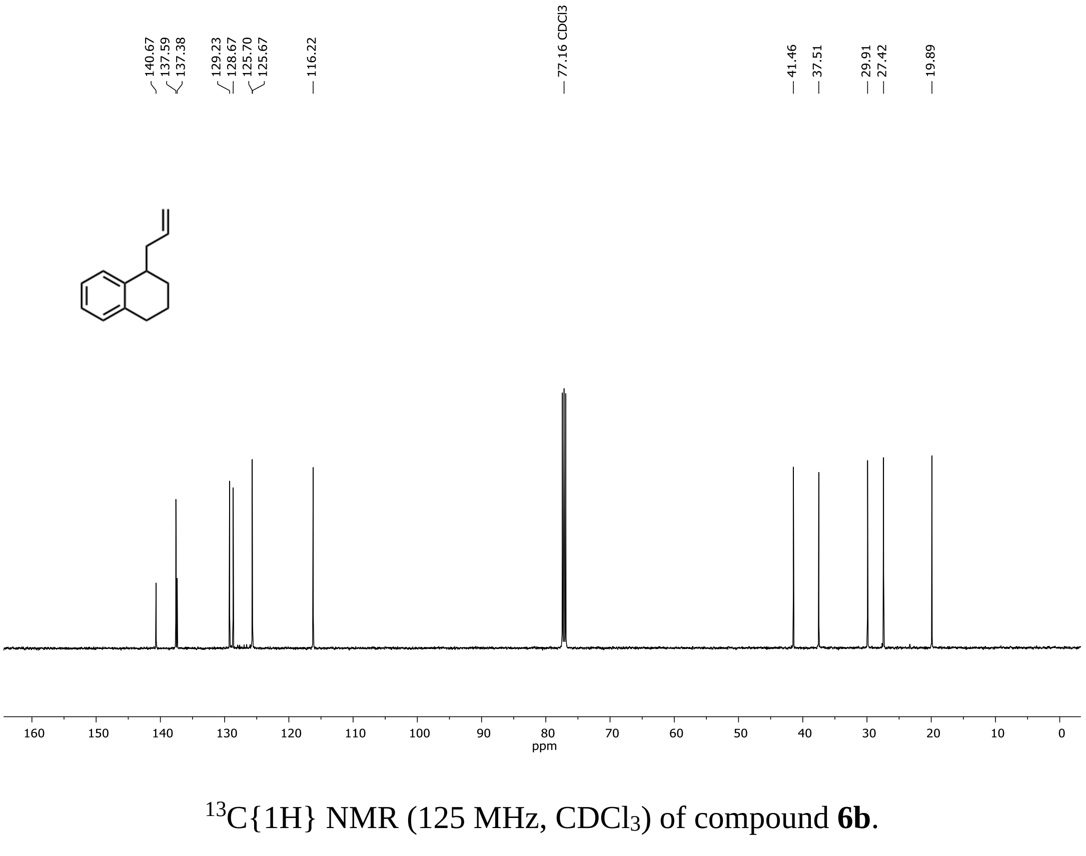

$^{13}C\{1H\}$ NMR (125 MHz, $CDCl_3$) of compound **6b**.

4-Naphthylpent-1-ene (6c)

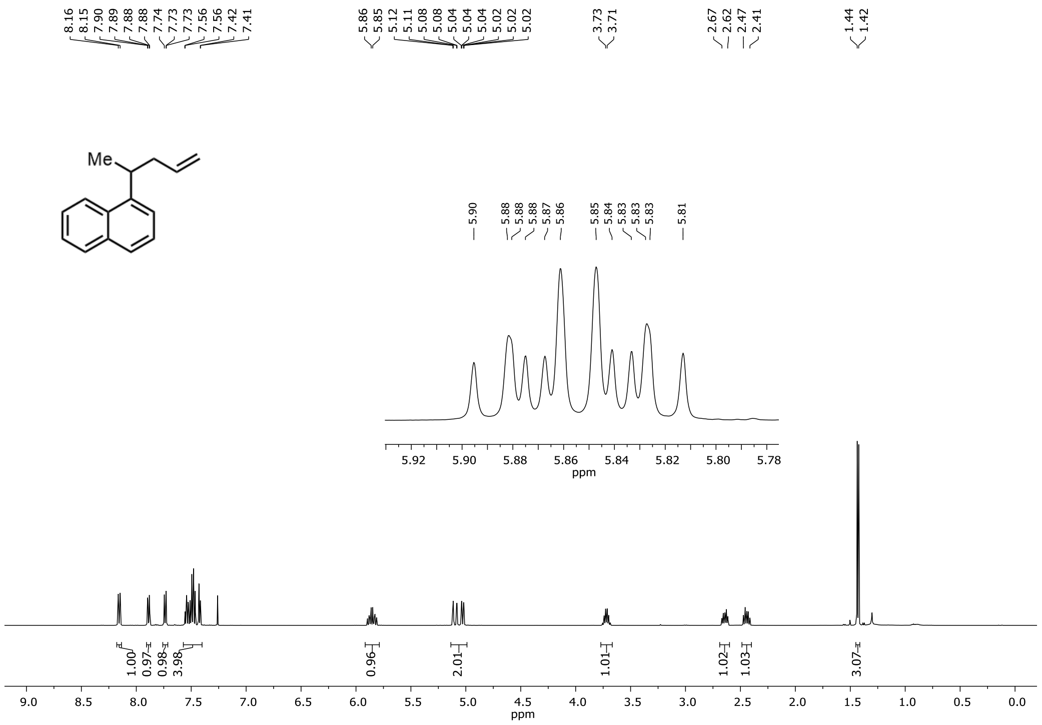

^{1}H NMR (500 MHz, $CDCl_3$) of compound **6c**.

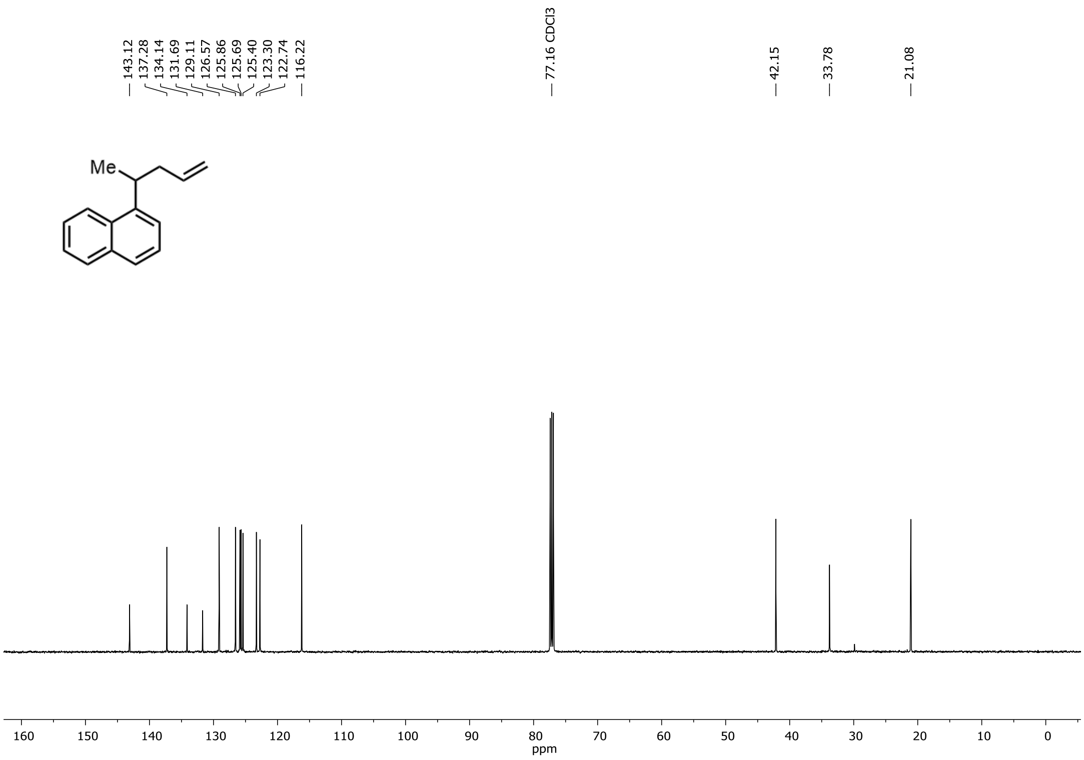

$^{13}C\{1H\}$ NMR (125 MHz, $CDCl_3$) of compound **6c**.

4-(4-Fluorobenzene)-pent-1-ene (6d)

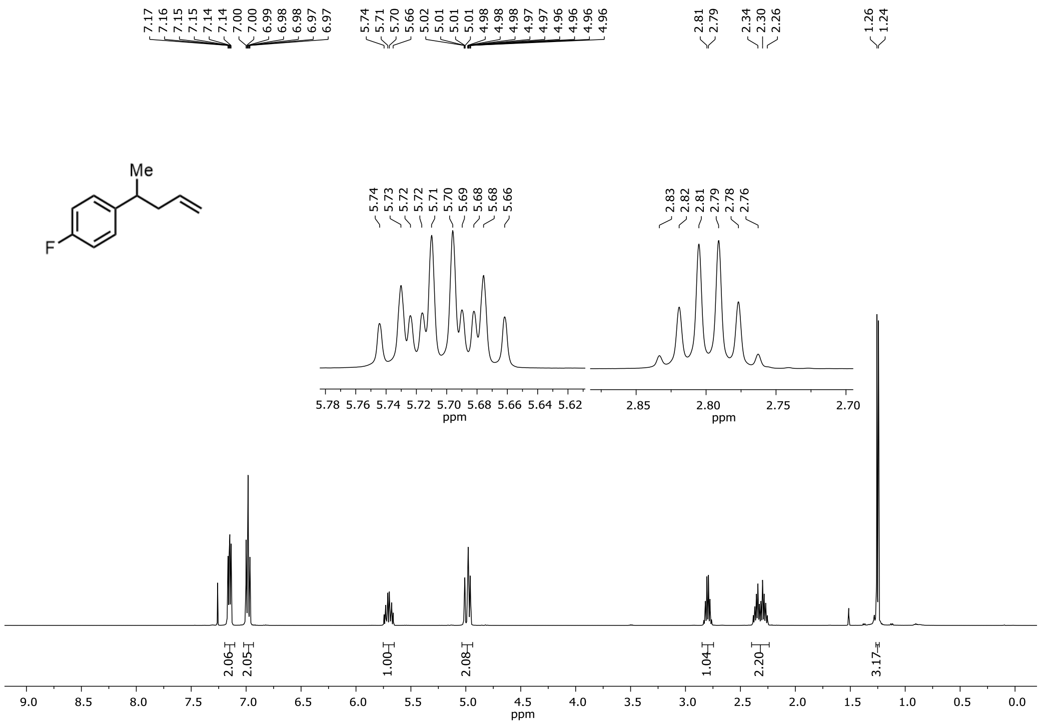

^{1}H NMR (500 MHz, $CDCl_3$) of compound **6d**.

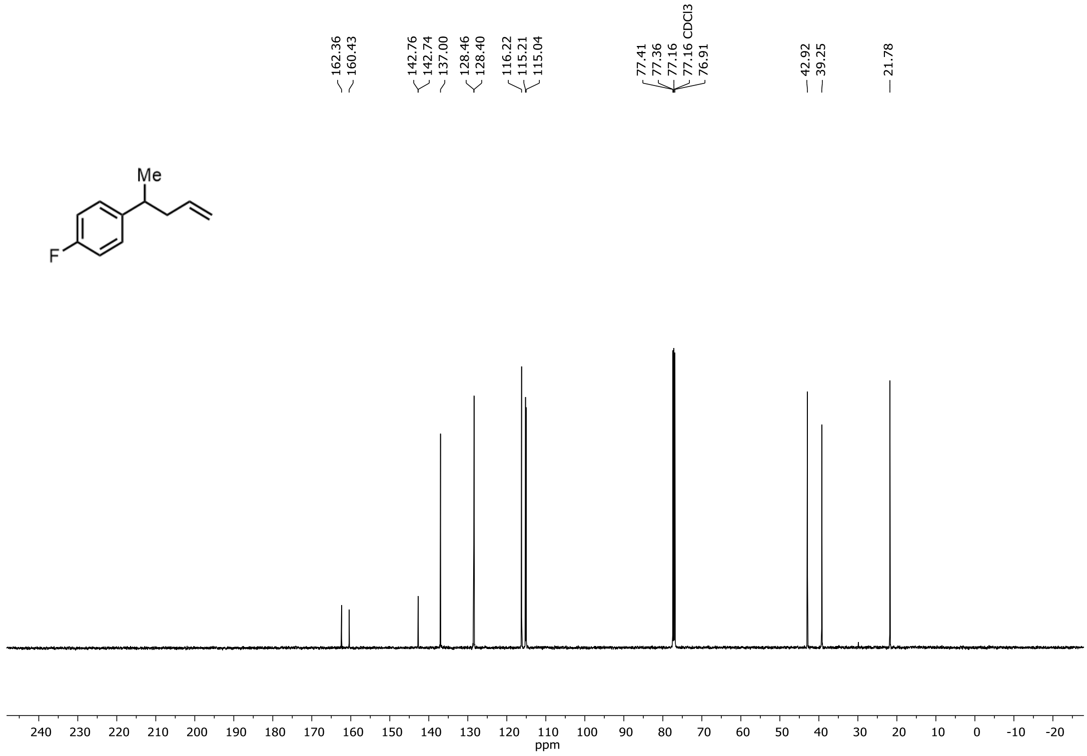

^{13}C{1H} NMR (125 MHz, $CDCl_3$) of compound **6d**.

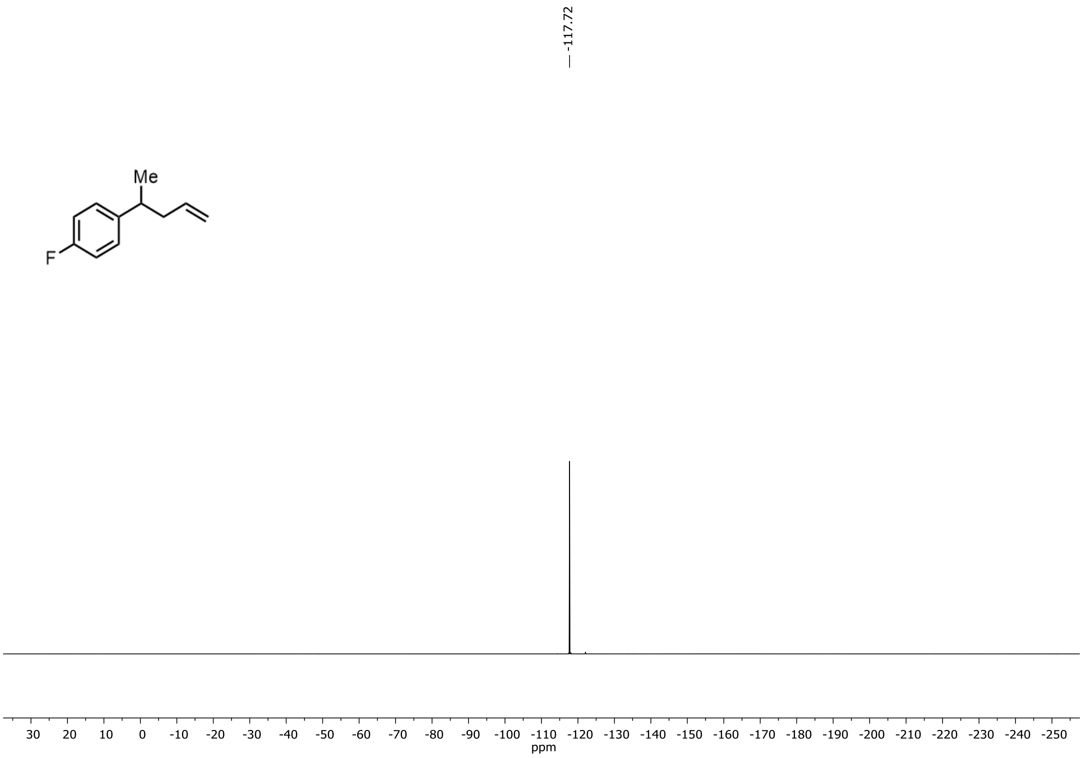

^{19}F NMR (470 MHz, $CDCl_3$) of compound **6d**.

4-(3,4,5-Trimethoxybenzene)-pent-1-ene (6e)

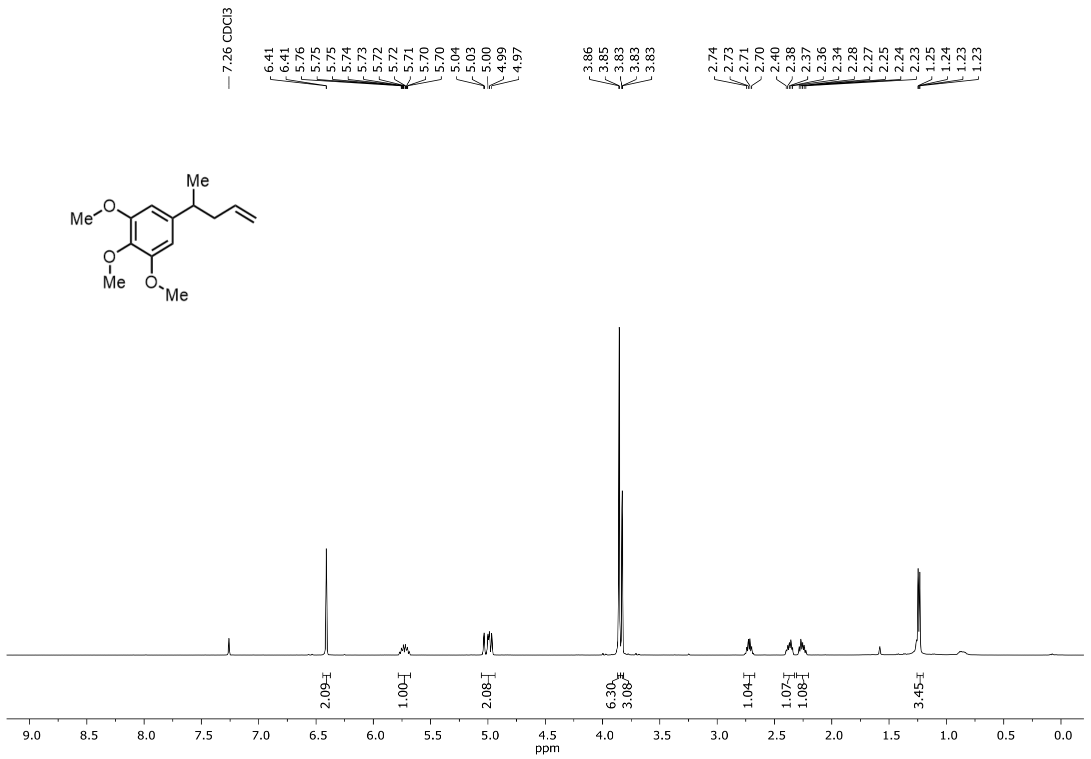

^{1}H NMR (500 MHz, $CDCl_3$) of compound **6e**.

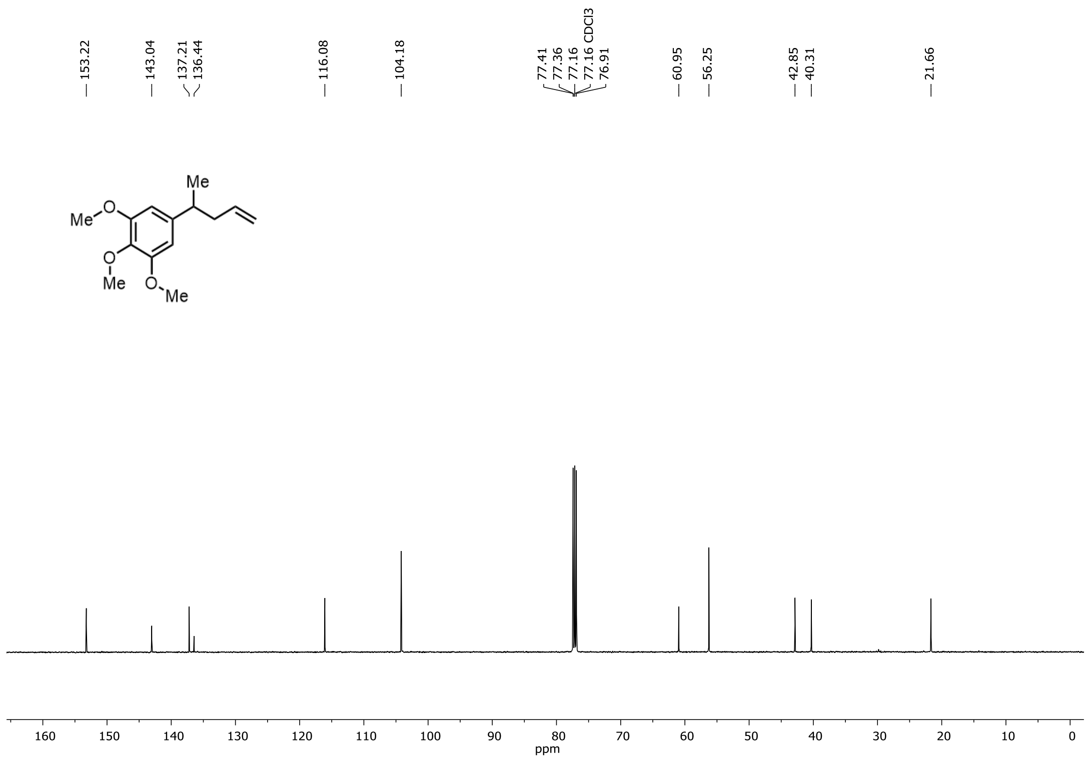

$^{13}C\{1H\}$ NMR (125 MHz, $CDCl_3$) of compound **6e**.

4,4-Diphenylbut-1-ene (6f)

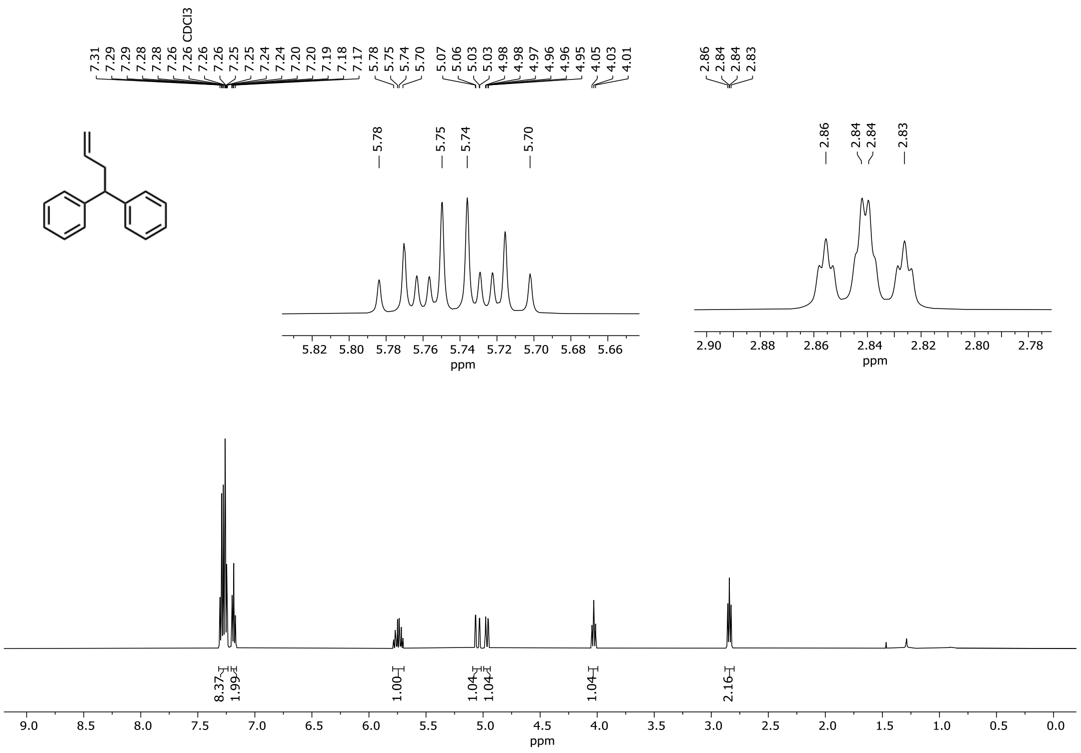

^{1}H NMR (500 MHz, $CDCl_3$) of compound **6f**.

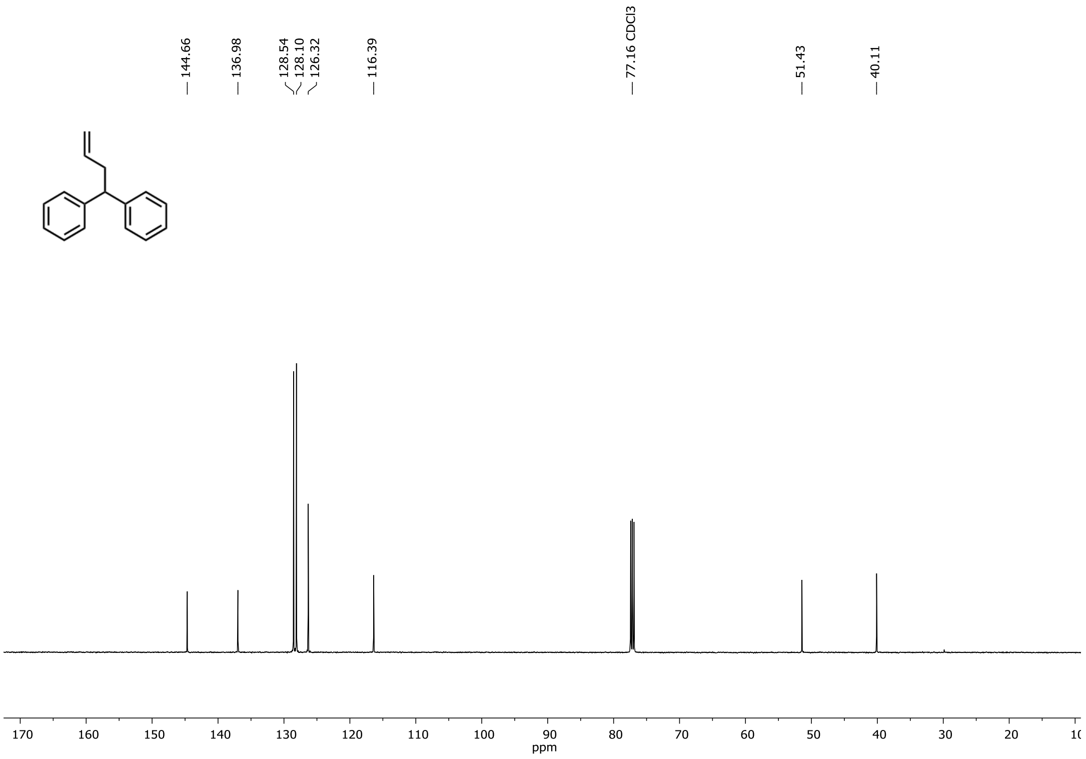

$^{13}C\{1H\}$ NMR (125 MHz, $CDCl_3$) of compound **6f**.

Di-(4-methoxyphenyl)-methane (9a)

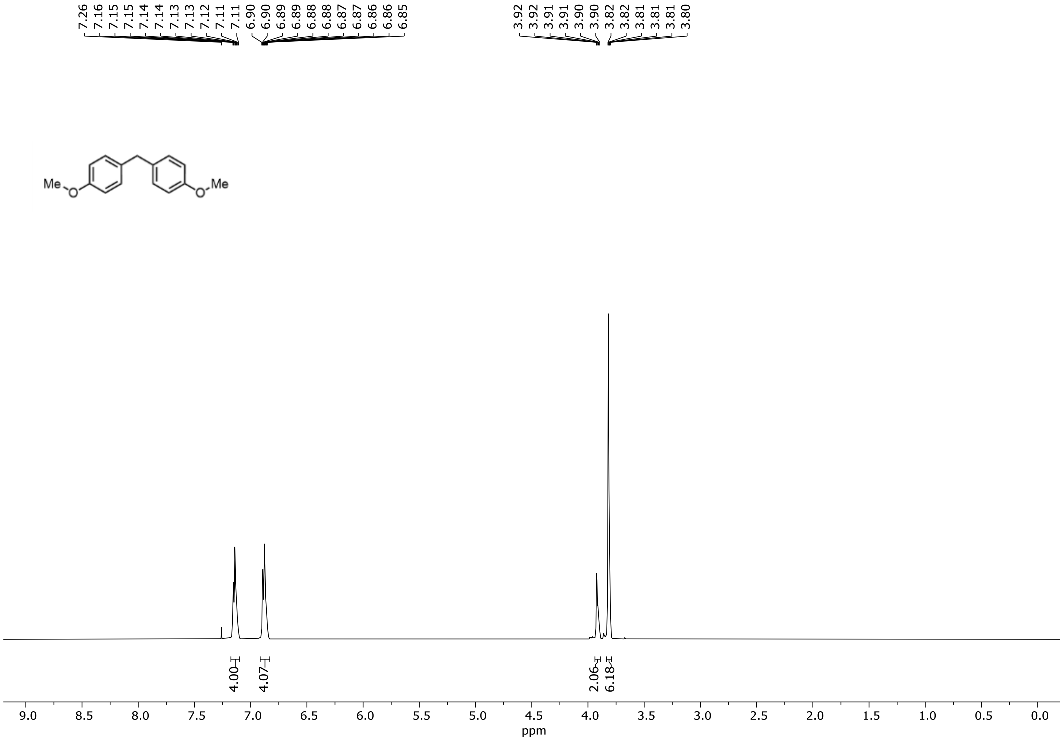

^{1}H NMR (500 MHz, $CDCl_3$) of compound **9a**.

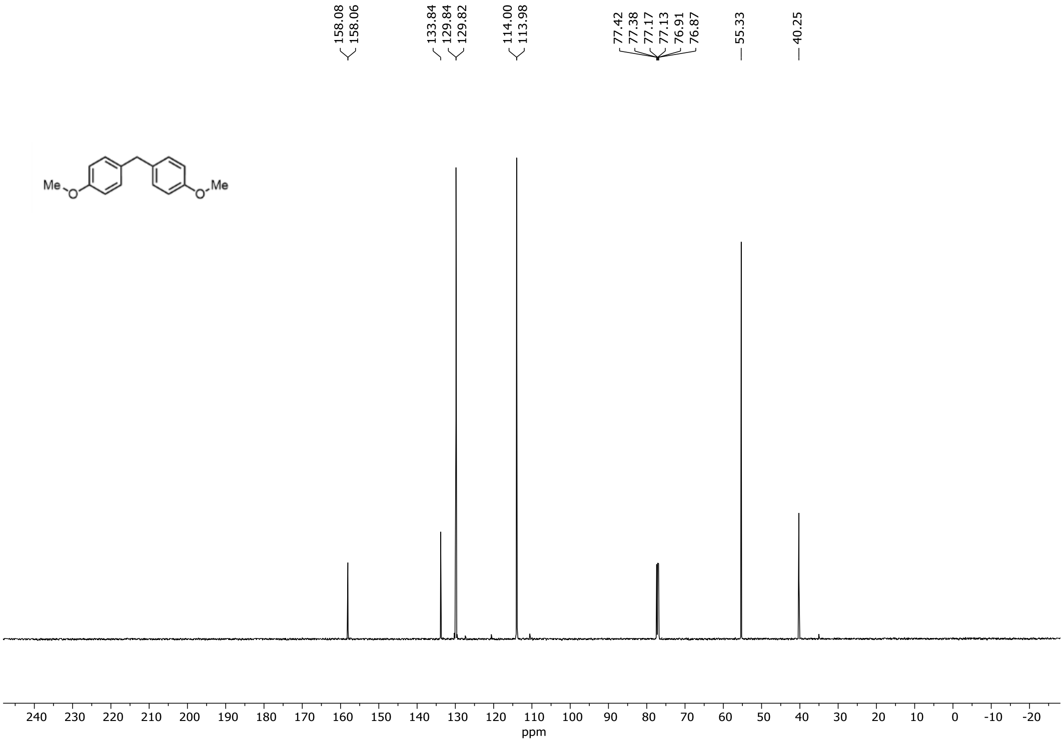

^{13}C{1H} NMR (125 MHz, $CDCl_3$) of compound **9a**.

2-(4-Methoxybenzyl)-1,3,5-trimethylbenzene (9b)

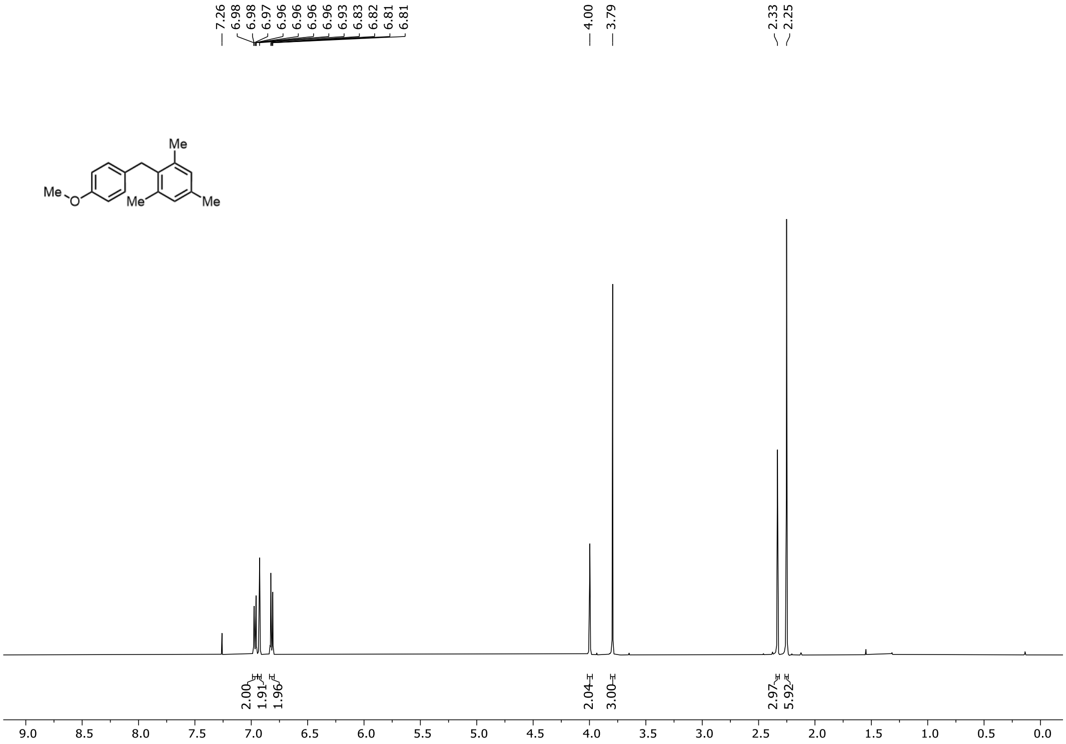

^{1}H NMR (500 MHz, $CDCl_3$) of compound **9b**.

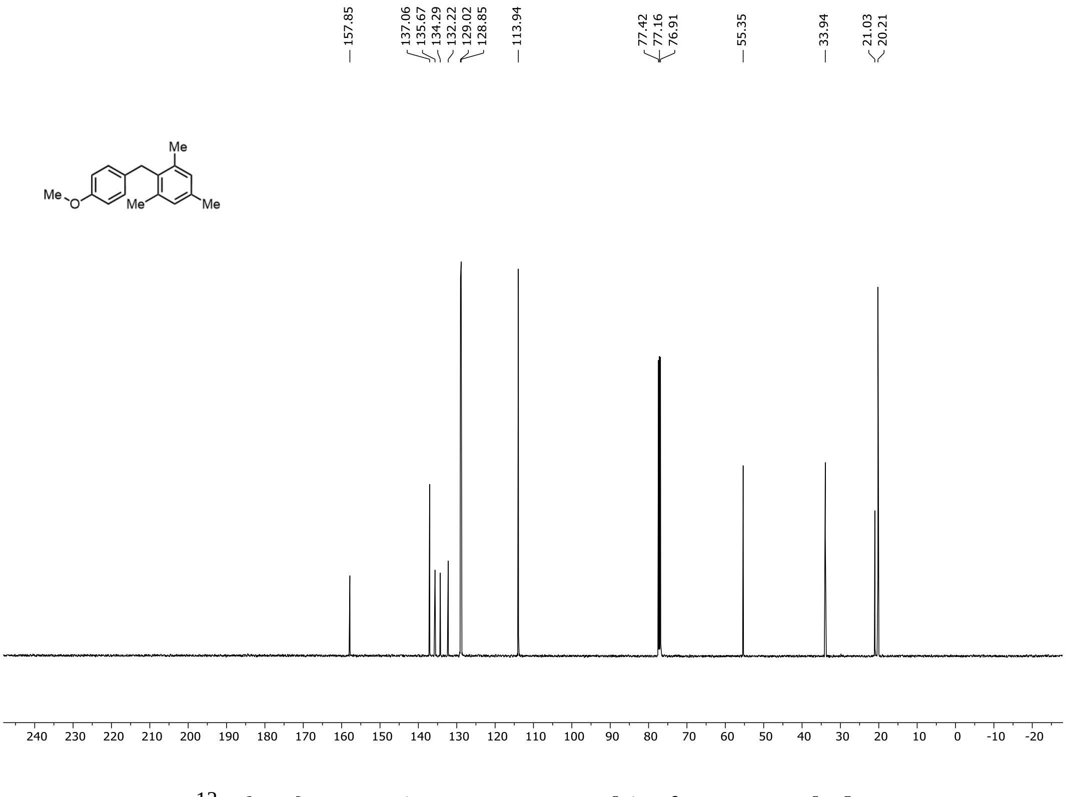

^{13}C{1H} NMR (125 MHz, $CDCl_3$) of compound **9b**.

3-(4-Methoxybenzyl)-2,4-pentadione (9c)

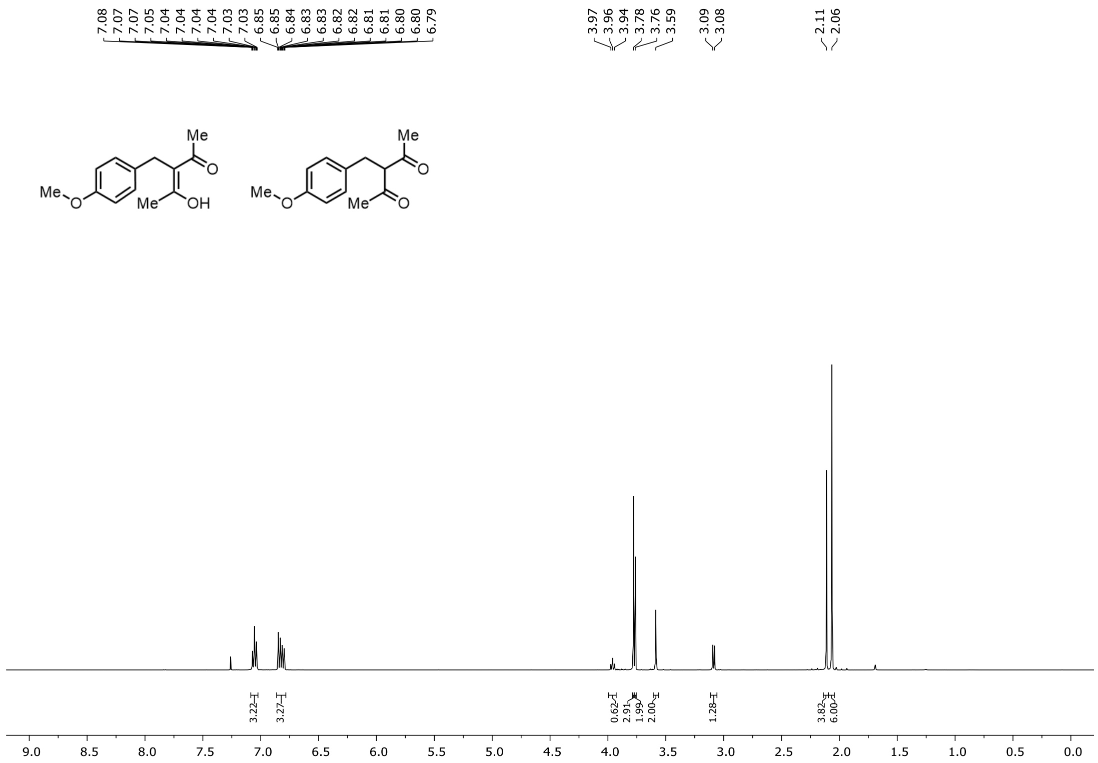

^{1}H NMR (500 MHz, $CDCl_3$) of compound **9c**.

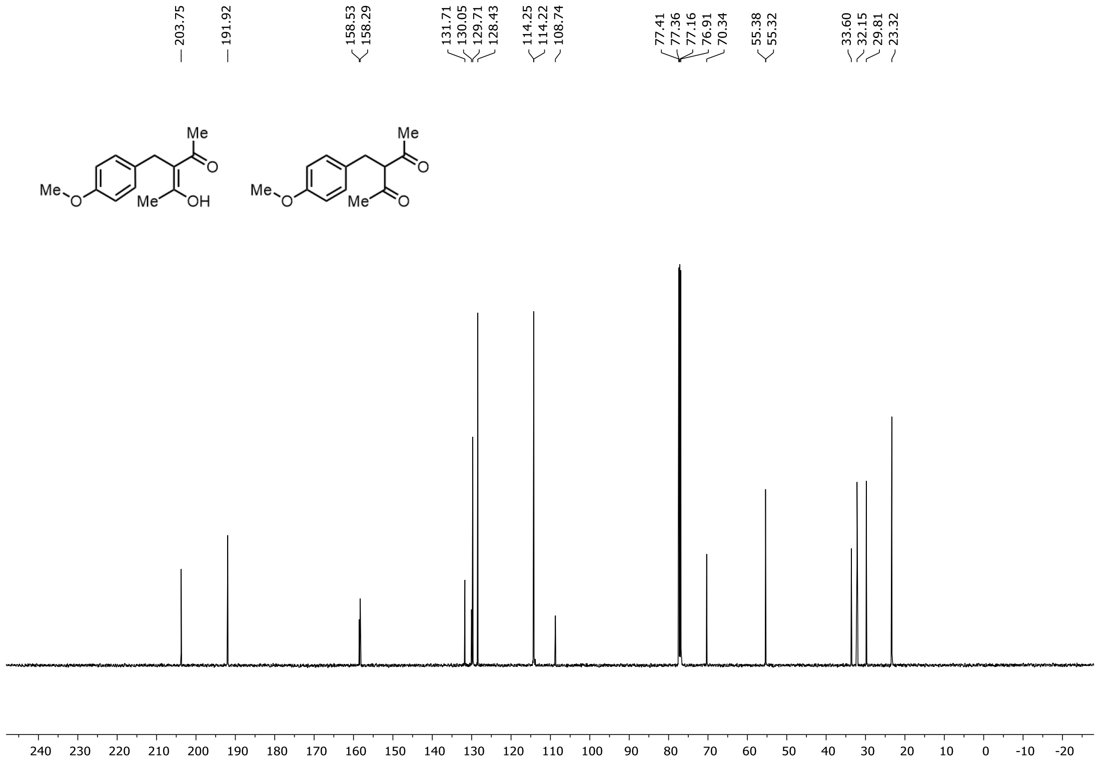

^{13}C{1H} NMR (125 MHz, $CDCl_3$) of compound **9c**.

4-(4-Methoxyphenyl)-2-methyl-1-butene (9d)

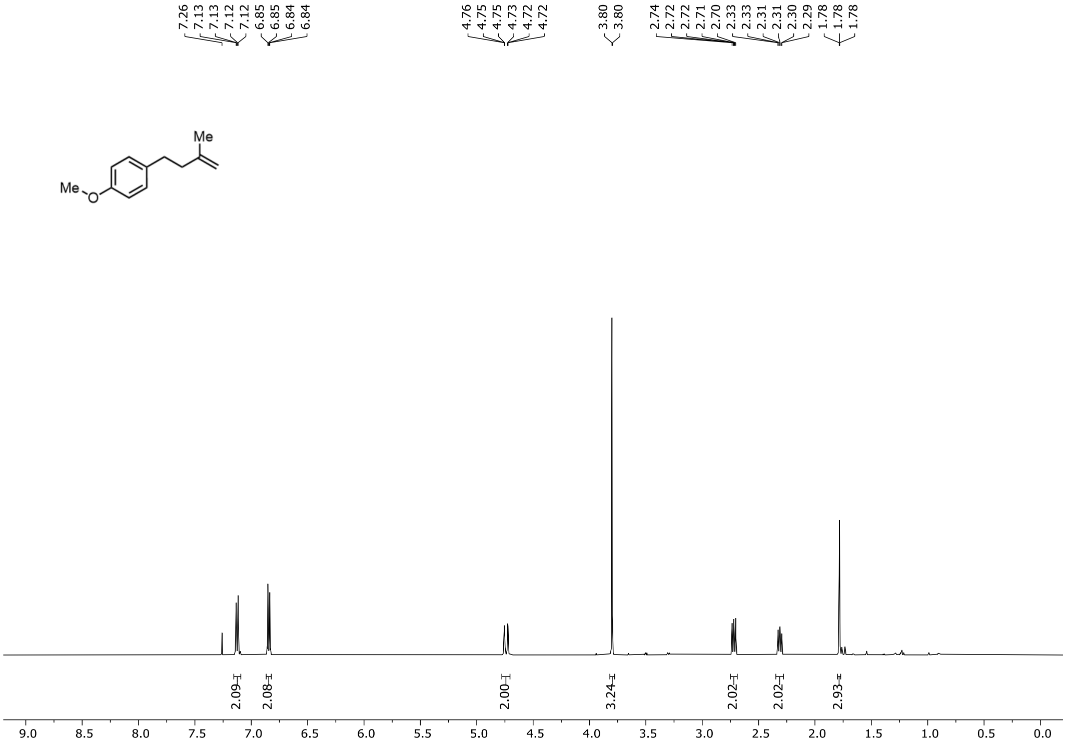

^{1}H NMR (500 MHz, $CDCl_3$) of compound **9d**.

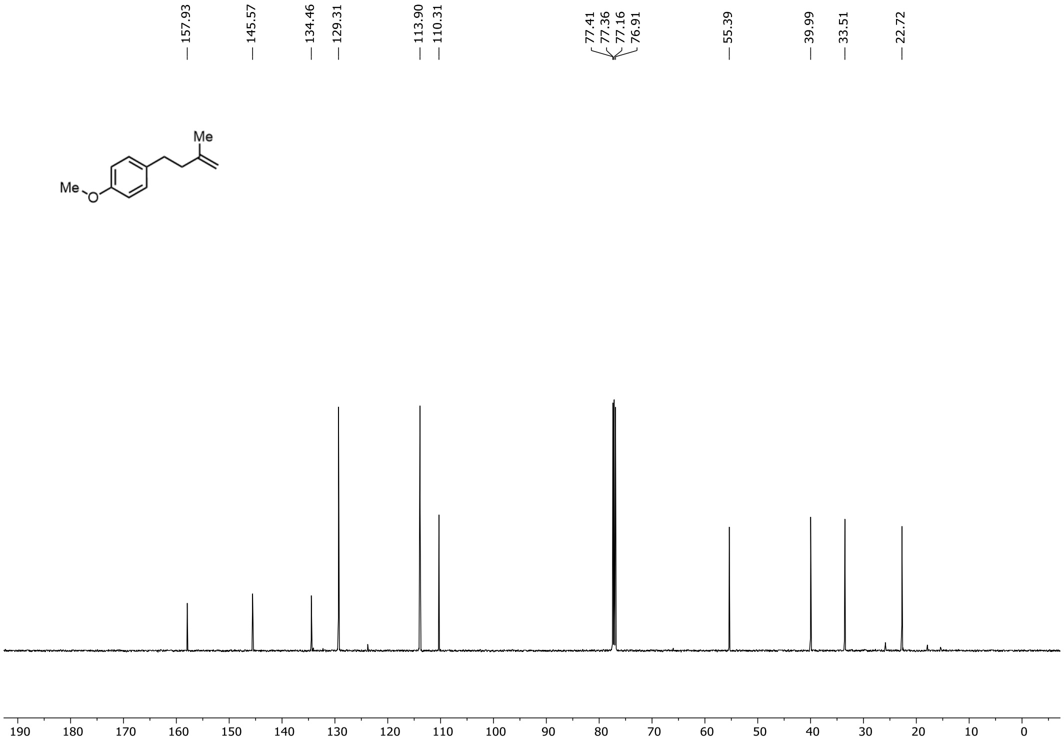

^{13}C{1H} NMR (125 MHz, $CDCl_3$) of compound **9d**.

3-(Methoxyphenyl)-2-methyl-1-phenyl-1-propanone (9e)

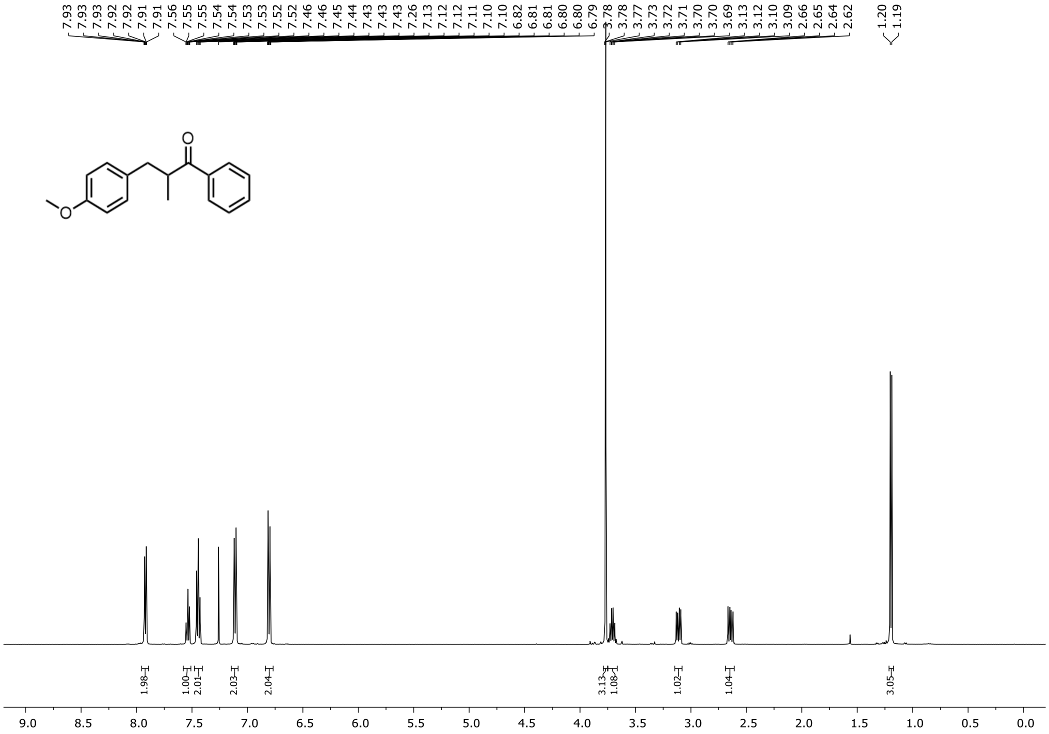

^{1}H NMR (500 MHz, $CDCl_3$) of compound **9e**.

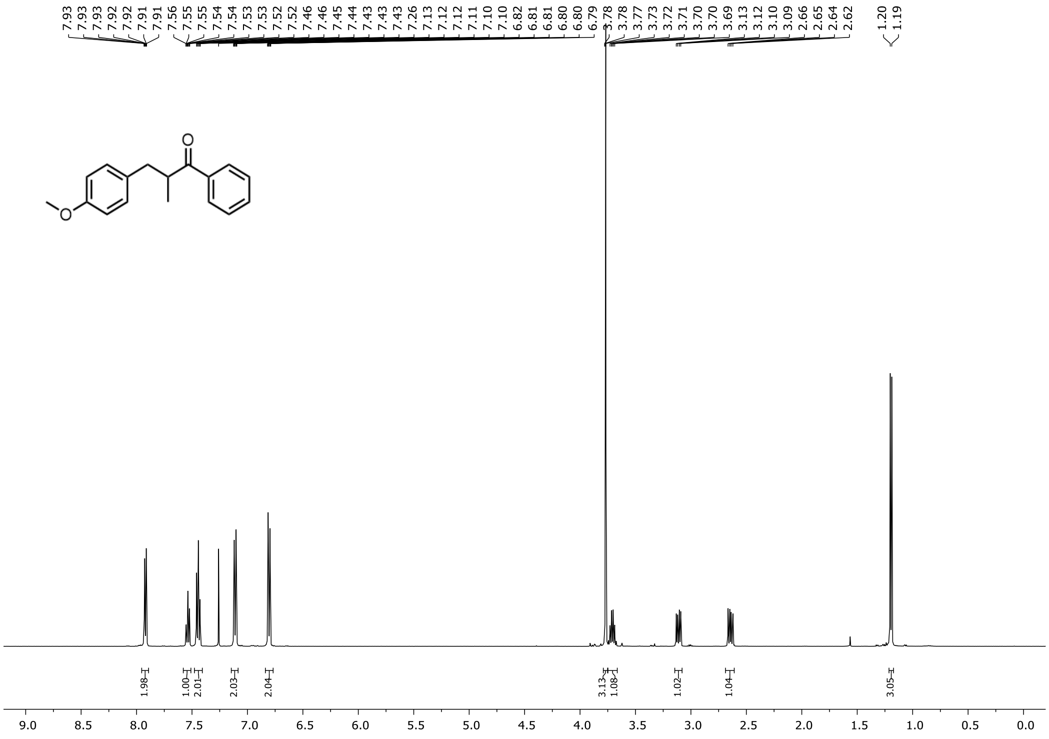

^{13}C{1H} NMR (125 MHz, $CDCl_3$) of compound **9e**.

4-(4-Methoxyphenyl)-2-butanone (9f)

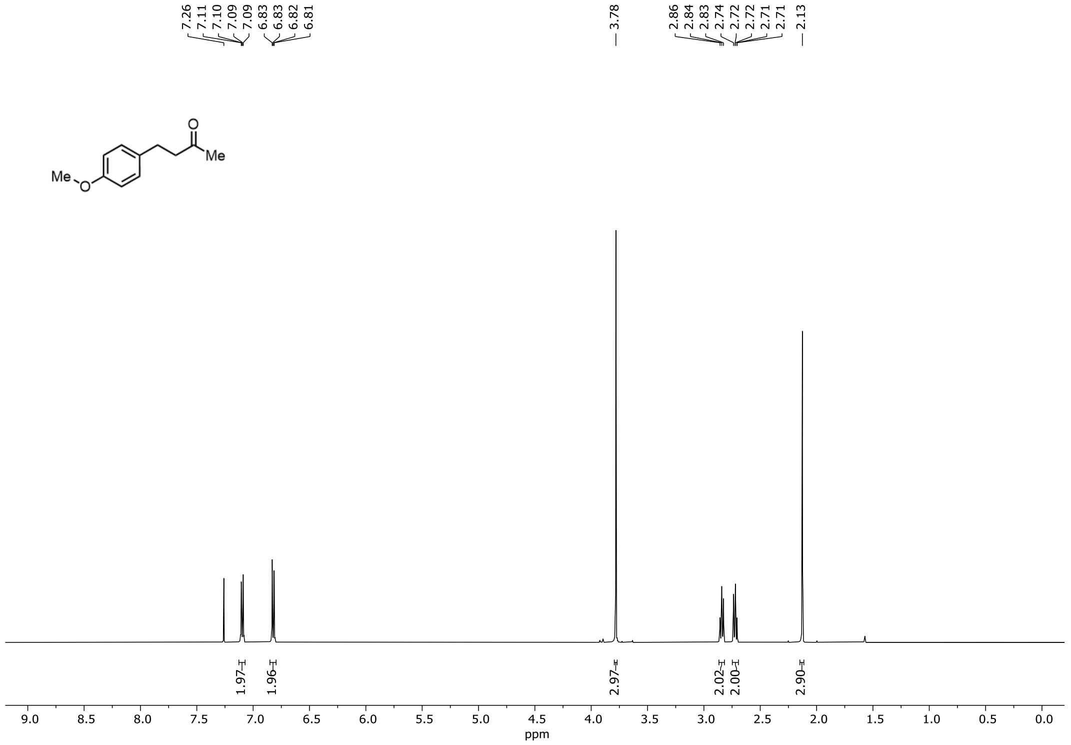

^{1}H NMR (500 MHz, $CDCl_3$) of compound **9f**.

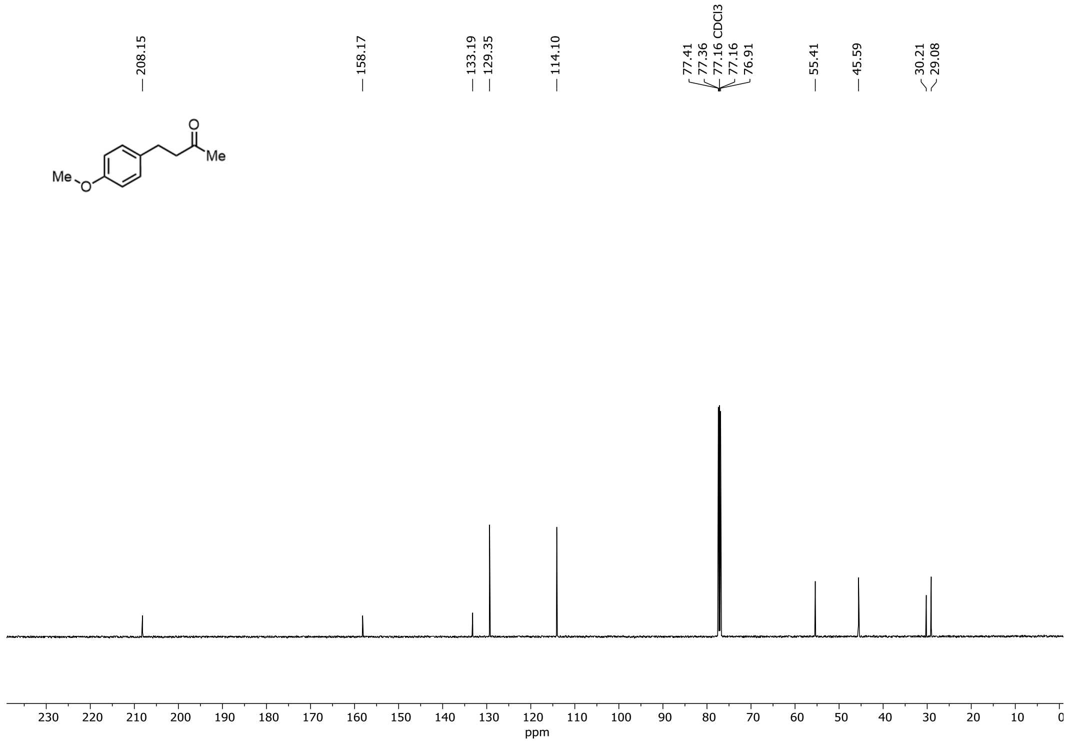

^{13}C{1H} NMR (125 MHz, $CDCl_3$) of compound **9f**.

1-Bromo-4-(1-ethoxybut-3-en)-benzene (11a)

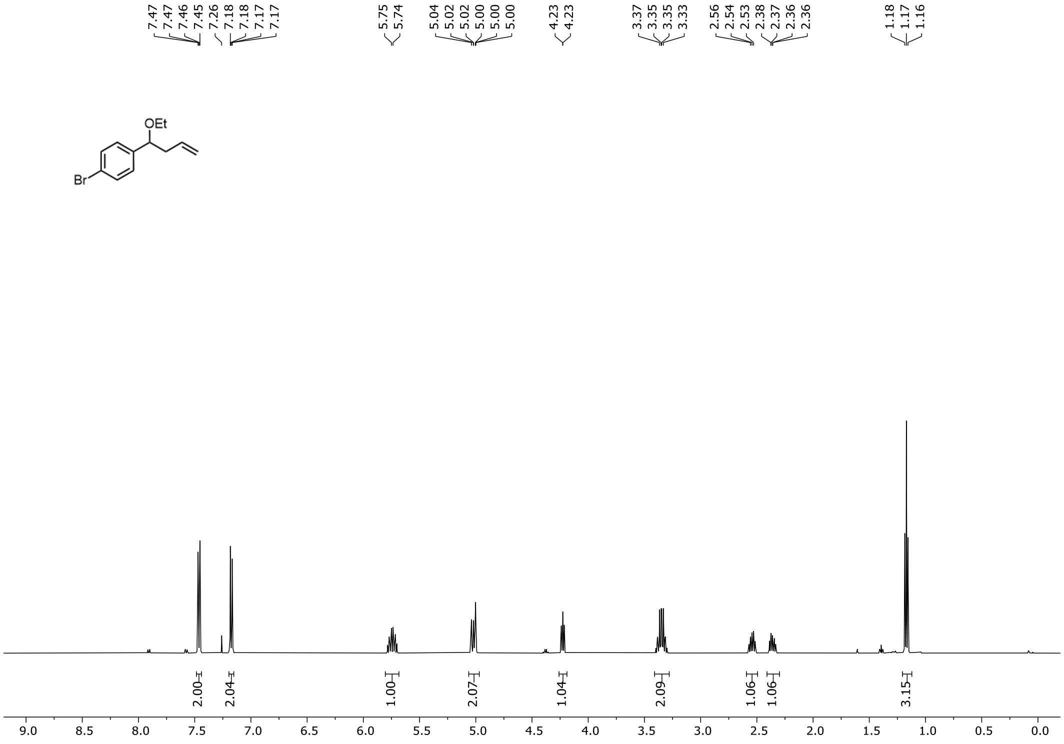

^{1}H NMR (500 MHz, $CDCl_3$) of compound **11a**.

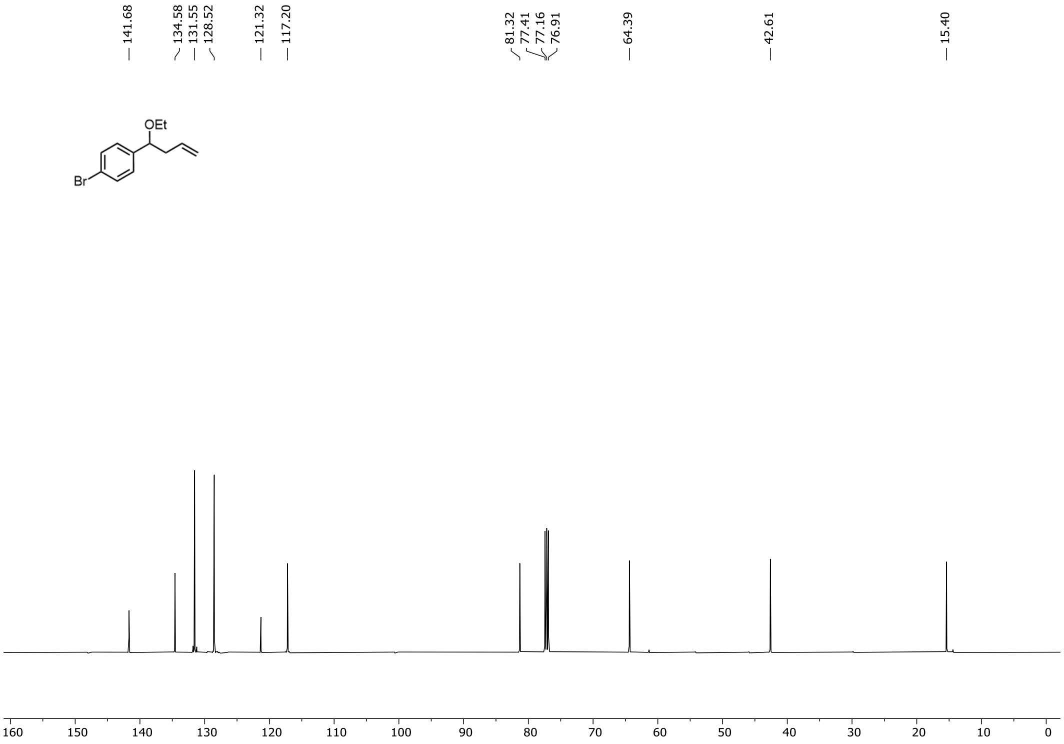

^{13}C{1H} NMR (125 MHz, $CDCl_3$) of compound **11a**.

1-(1-Methoxybut-3-ene)-4-nitrobenzene (11b)

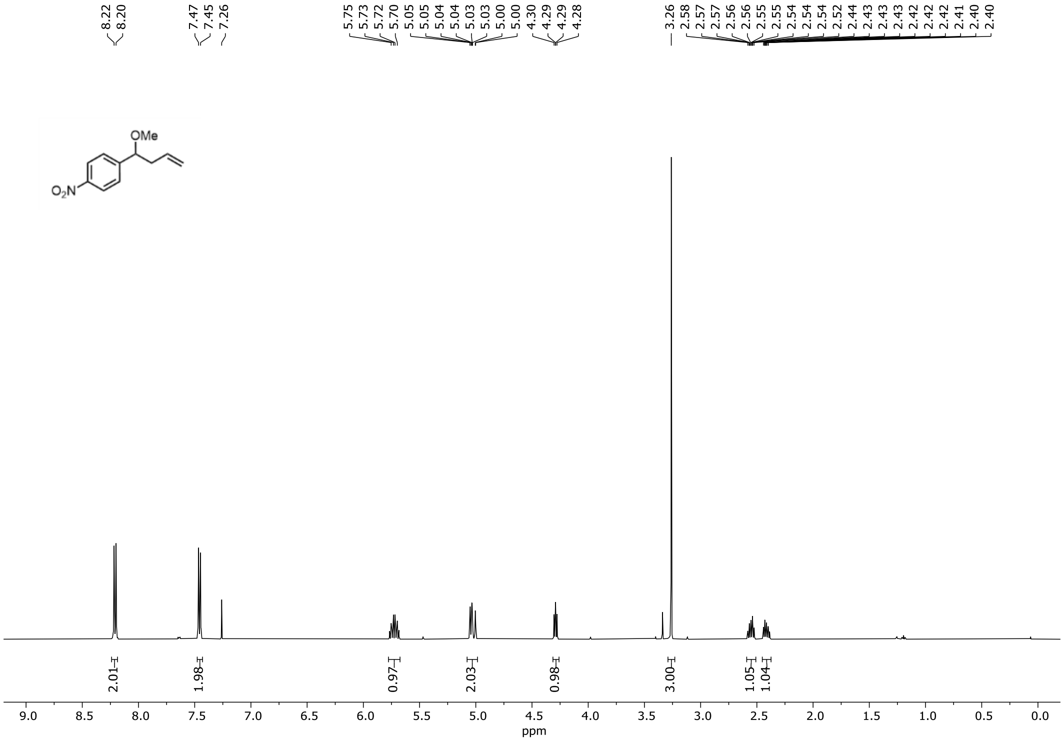

^{1}H NMR (500 MHz, $CDCl_3$) of compound **11b**.

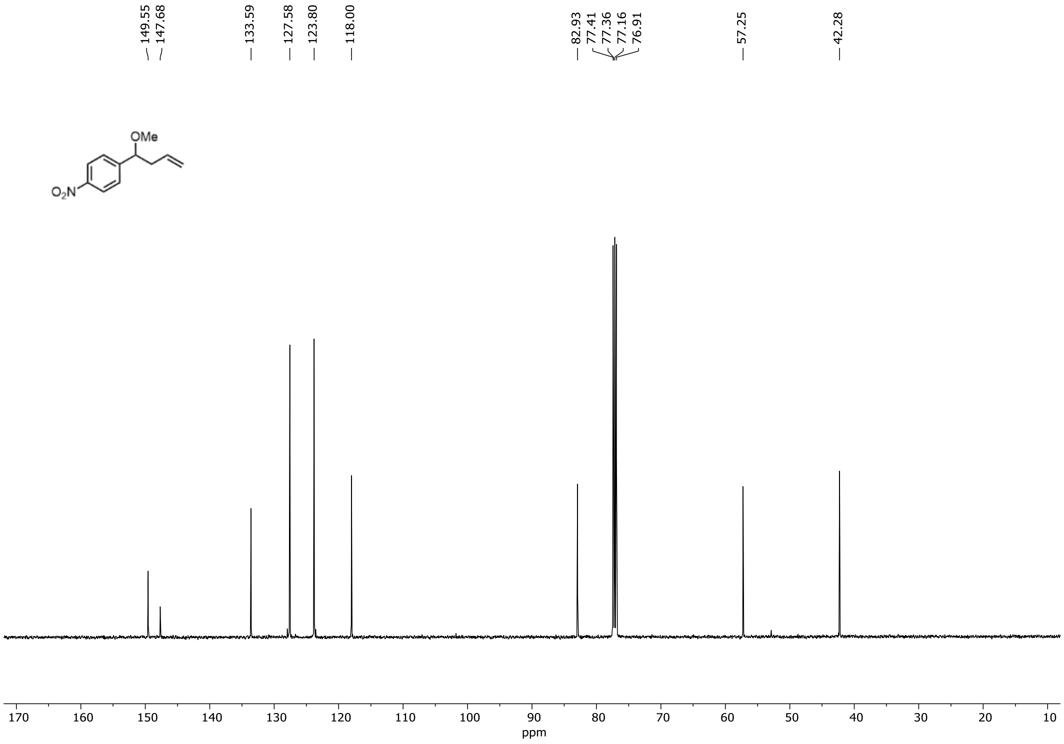

^{13}C NMR (125 MHz, $CDCl_3$) of compound **11b**.

4-Methoxy-5-phenyl-1-pentene (11c)

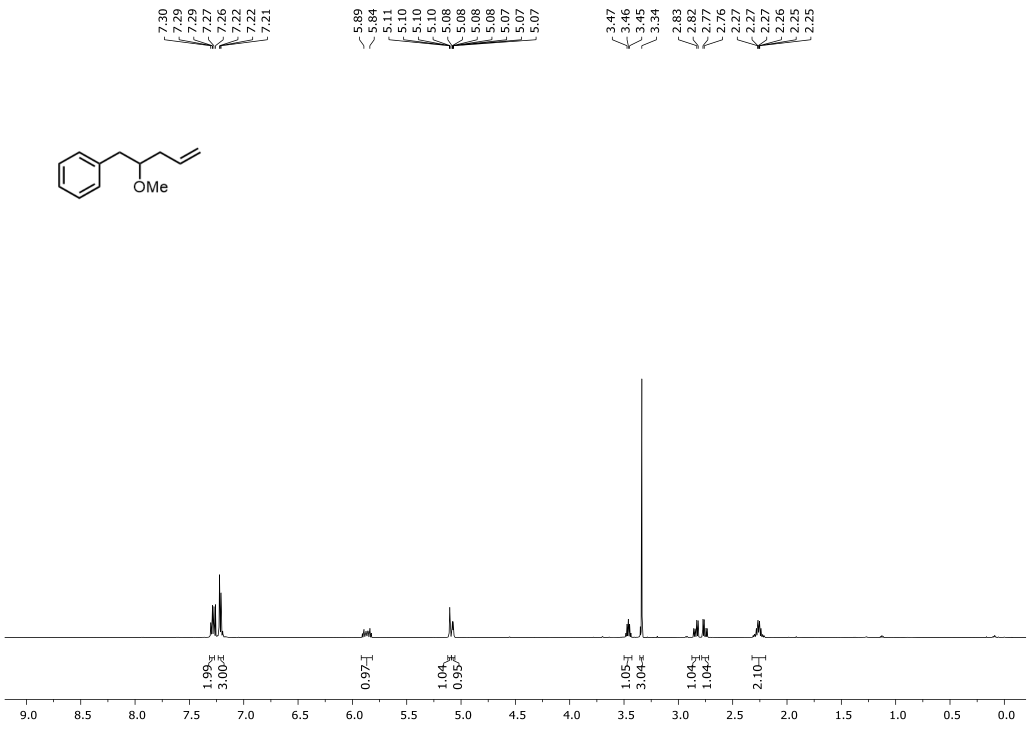

^{1}H NMR (500 MHz, $CDCl_3$) of compound **11c**.

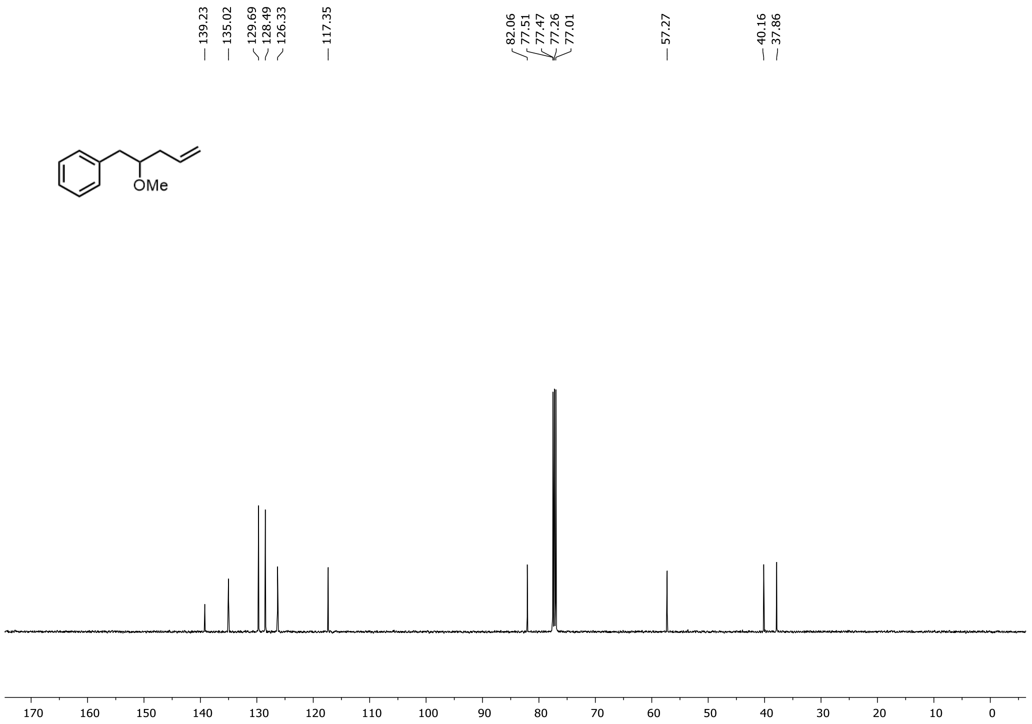

$^{13}C\{1H\}$ NMR (125 MHz, $CDCl_3$) of compound **11c**.

4-(4-Methoxybenzene)-hepta-1,6-diene (12)

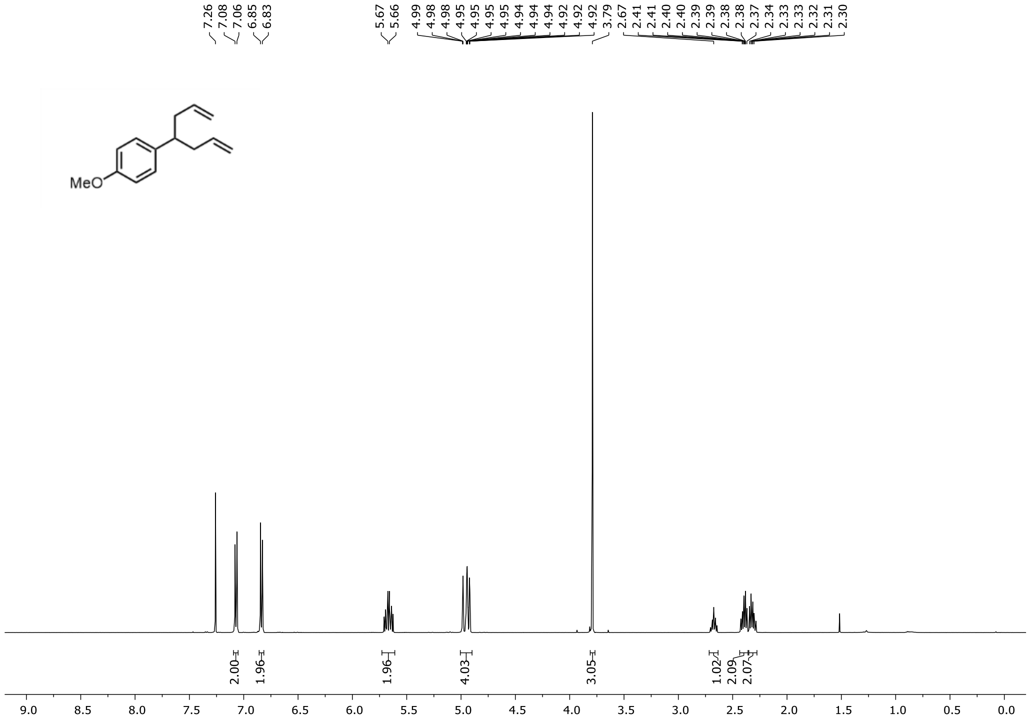

^{1}H NMR (500 MHz, $CDCl_3$) of compound **12**.

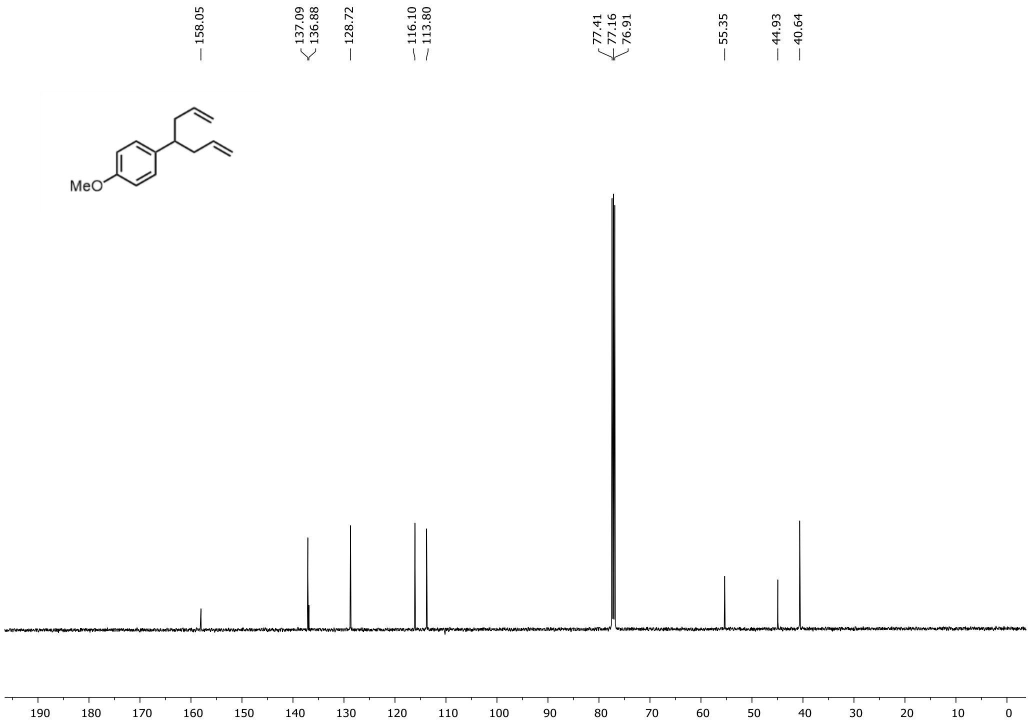

^{13}C{1H} NMR (125 MHz, $CDCl_3$) of compound **12**.

5 References

[1] B. Ciszek, I. Fleischer, *Chem. Eur. J.* **2018**, *24*, 12259.

[2] X. Fan, X.-M. Cui, Y.-H. Guan, L.-A. Fu, H. Lv, K. Guo, H.-B. Zhu, *Eur. J. Org. Chem.* **2014**, 498.

[3] D. Marković, W. A. Tchawou, I. Novosjolova, S. Laclef, D. Stepanovs, M. Turks, P. Vogel, *Chem. Eur. J.* **2016**, *22*, 4196.

[4] R. Hayashi, A. Shimizu, J.-i. Yoshida, *J. Am. Chem. Soc.* **2016**, *138*, 8400.

[5] G. Onodera, E. Yamamoto, S. Tonegawa, M. Iezumi, R. Takeuchi, *Adv. Synth. Catal.* **2011**, *353*, 2013.

[6] T. Saito, Y. Nishimoto, M. Yasuda, A. Baba, *J. Org. Chem.* **2006**, *71*, 8516.

[7] L. Bering, K. Jeyakumar, A. P. Antonchick, *Org. Lett.* **2018**, *20*, 3911.

[8] P. Biswal, S. Samser, S. K. Meher, V. Chandrasekhar, K. Venkatasubbaiah, *Adv. Synth. Catal.* **2022**, *364*, 413.

[9] A. Boffi, S. Cacchi, P. Ceci, R. Cirilli, G. Fabrizi, A. Prastaro, S. Niembro, A. Shafir, A. Vallribera, *ChemCatChem* **2011**, *3*, 347.

[10] L.-G. Xie, J. Rogers, I. Anastasiou, J. A. Leitch, D. J. Dixon, *Org. Lett.* **2019**, *21*, 6663.

[11] S. Ito, A. Hayashi, H. Komai, H. Yamaguchi, Y. Kubota, M. Asami, *Tetrahedron* **2011**, *67*, 2081.

[12] T. P. Blaisdell, T. C. Caya, L. Zhang, A. Sanz-Marco, J. P. Morken, *J. Am. Chem. Soc.* **2014**, *136*, 9264.

[13] R. Tomifuji, S. Masuda, T. Kurahashi, S. Matsubara, *Org. Lett.* **2019**, *21*, 3834.
